# Genome-Wide Association Mapping for Tolerance to Preharvest Sprouting and Low Falling Numbers in Wheat

**DOI:** 10.3389/fpls.2018.00141

**Published:** 2018-02-14

**Authors:** Shantel A. Martinez, Jayfred Godoy, Meng Huang, Zhiwu Zhang, Arron H. Carter, Kimberly A. Garland Campbell, Camille M. Steber

**Affiliations:** ^1^Molecular Plant Sciences, Washington State University, Pullman, WA, United States; ^2^Department of Crop and Soil Sciences, Washington State University, Pullman, WA, United States; ^3^USDA-ARS Wheat Health, Genetics, and Quality Research Unit, Washington State University, Pullman, WA, United States

**Keywords:** preharvest sprouting, falling number, seed dormancy, wheat, association mapping

## Abstract

Preharvest sprouting (PHS), the germination of grain on the mother plant under cool and wet conditions, is a recurring problem for wheat farmers worldwide. α-amylase enzyme produced during PHS degrades starch resulting in baked good with poor end-use quality. The Hagberg-Perten Falling Number (FN) test is used to measure this problem in the wheat industry, and determines how much a farmer's wheat is discounted for PHS damage. PHS tolerance is associated with higher grain dormancy. Thus, breeding programs use germination-based assays such as the spike-wetting test to measure PHS susceptibility. Association mapping identified loci associated with PHS tolerance in U.S. Pacific Northwest germplasm based both on FN and on spike-wetting test data. The study was performed using a panel of 469 white winter wheat cultivars and elite breeding lines grown in six Washington state environments, and genotyped for 15,229 polymorphic markers using the 90k SNP Illumina iSelect array. Marker-trait associations were identified using the FarmCPU R package. Principal component analysis was directly and a kinship matrix was indirectly used to account for population structure. Nine loci were associated with FN and 34 loci associated with PHS based on sprouting scores. None of the *QFN.wsu* loci were detected in multiple environments, whereas six of the 34 *QPHS.wsu* loci were detected in two of the five environments. There was no overlap between the QTN detected based on FN and PHS, and there was little correlation between the two traits. However, both traits appear to be PHS-related since 19 of the 34 *QPHS.wsu* loci and four of the nine *QFN.wsu* loci co-localized with previously published dormancy and PHS QTL. Identification of these loci will lead to a better understanding of the genetic architecture of PHS and will help with the future development of genomic selection models.

## Introduction

Rainy conditions before harvest can cause mature grain to initiate germination while still on the mother plant (Rodríguez et al., 2015). This problem, called preharvest sprouting (PHS), occurs in many cereal crops such as wheat (*Triticum aestivum* L.), barley (*Hordeum vulgare* L.), and sorghum (*Sorghum bicolor*) (Paterson and Sorrells, 1990; Gualano et al., 2007; Ullrich et al., 2009). Germination is associated with α-amylase enzyme induction in order to mobilize starch reserves for use by the growing embryo (Clarke et al., 1984). This α-amylase induction during PHS in wheat grain leads to problems with poor end-use quality due to starch degradation. Thus, sprouted wheat grain is discounted in the marketplace.

The degree of PHS tolerance is associated with multiple environmental and genetic factors. Grain dormancy can account for up to 60% of variation in PHS tolerance, although spike morphology and epicuticular waxes are also associated with PHS response (King, 1984; King and Richards, 1984; DePauw and McCaig, 1991; King and von Wettstein-Knowles, 2000). Dormant seeds cannot germinate under favorable environmental conditions (light, moisture, and temperature) (Bewley and Black, 1994). Seeds are most dormant at physiological maturity, and then lose dormancy through a period of dry storage called after-ripening (Finkelstein et al., 2008). Dormancy can also be broken by moist chilling (called cold stratification) or seed coat scarification (Paterson et al., 1989; Finkelstein et al., 2008). The degree of wheat grain dormancy and PHS tolerance depends on environmental conditions both before and after the grain reaches physiological maturity. Grain dormancy is higher when the mother plant is exposed to cooler conditions during the maturation phase of grain development (Nakamura et al., 2011). Conversely, cold stratification of mature grain breaks dormancy. If rain and cold temperatures occur after physiological maturity, seed dormancy can be broken through cold stratification and grain is more likely to sprout. Thus, cold temperatures have opposite effects depending on whether they occur prior to or after the maturation date. Because seed dormancy is also broken through dry after-ripening, wheat also becomes more likely to sprout the longer unharvested mature grain stands dry in the field before it rains (Gerjets et al., 2010). Thus, variation in PHS tolerance also depends on when the rain occurred relative to maturation date. Higher PHS tolerance is associated with genetic loci that increase grain dormancy including red kernel color and the synthesis or response to the dormancy-inducing hormone ABA (abscisic acid) (Walker-Simmons, 1987; Flintham, 2000; Warner et al., 2000; Schramm et al., 2010; Himi et al., 2011; Jaiswal et al., 2012; Kulwal et al., 2012; Martinez et al., 2016). Since lack of red kernel color reduces dormancy, other genetic mechanisms supporting PHS tolerance must be identified and selected in wheat with white kernels.

The spike-wetting test is often used to assess PHS tolerance in breeding programs because it examines multiple variables affecting PHS (Paterson et al., 1989). In order to control for variation due to maturity date, intact spikes are harvested from the field at physiological maturity and allowed to dry after-ripen for the same number of days (5–14 days) before placing them under a greenhouse misting system. The use of intact spikes takes into account the effect of awns, erectness of the spike, gloom tightness, and head type (Pool and Patterson, 1958; Ibrahim, 1966; Hong, 1979; King and Richards, 1984). Spikes are assigned a sprouting score using a scale based on visible germination and post-germinative growth throughout the spike (McMaster and Derera, 1976). The sprouting scores of McMaster and Derera (1976) actually reflect three biological stages: (1) initial germination or the first appearance of roots (scores 1-5), (2) coleoptile emergence (scores 6-8), and (3) seedling growth (scores 9-10). Other methods for assessing PHS tolerance include plating assays to assess degree of seed dormancy, the Falling Numbers test (see below), and variations on the spike-wetting test (Paterson et al., 1989; Kumar et al., 2009; Zhang et al., 2014; Jiménez et al., 2016; Zhou et al., 2017).

Damage due to α-amylase induction from PHS is measured in the wheat industry using the Hagberg-Perten Falling Numbers (FN) test (Perten, 1964). During the FN test, a slurry of wheat meal and water is mixed while being heated to 100°C. Then the FN machine measures the time in seconds (sec) that it takes for a stirrer to fall through the slurry. The higher the α-amylase level, the thinner the slurry, allowing the stirrer to fall faster resulting in a lower FN. If the FN is below 300 s, then the farmer receives significantly less money for his/her grain. Because some studies have shown a significant correlation between FN and sprouting scores, one might expect spike-wetting tests and FN to map similar PHS tolerance loci (reviewed by DePauw et al., 2012). However, this has not been directly tested.

Here we present a genome-wide association mapping study (GWAS) for PHS tolerance in white wheat based on sprouting scores and FN. A large panel of 469 white wheat lines representing six northwestern U.S. breeding programs was examined over multiple environments in order to characterize the genetic architecture of PHS tolerance/susceptibility. The goal was to identify quantitative trait nucleotides (QTN) associated with PHS tolerance in white wheat breeding programs, while examining the phenotypic connection between FN and spike-wetting test scores.

## Materials and methods

### Plant materials

This study used a mapping panel of 469 winter wheat accessions, consisting of advanced soft white breeding lines and cultivars from US Pacific Northwest breeding programs (Supplementary Table 1; Supplementary Figure 1). The accessions included 36% club (*T. aestivum* ssp *compactum*) genotypes with compact spike morphology and 64% soft white genotypes with lax spike morphology. The same panel was recently analyzed for soil acidity, aluminum tolerance, Cephalosporium stripe resistance, and stripe rust resistance (Froese and Carter, 2016; Froese et al., 2016; Liu et al., 2017).

### Field research environments

The mapping panel was grown as 1.5 m long headrows at Central Ferry, WA in 2014, 2015, and 2016 (C14, C15, C16), or as 8 m^2^ plots at the Washington State University Spillman Agronomy Farm in Pullman, WA in 2014, 2015, and 2016 (P14, P15, P16) using recommended agronomic practices for those locations. The panel was also grown as headrows in Pullman, WA in 2013 (P13). Heading dates from the Pullman 2014 environment were determined after 50% of the plot reached full spike emergence from the boot. Table 1 lists the planting dates and harvest dates for each environment. Spikes were harvested at physiological maturity, right after the peduncle turned yellow, for the spike-wetting test (see below) only in Pullman 2014, 2015, and 2016 and Central 2014 and 2015.

**Table 1:** Environments tested for preharvest sprouting traits. For the **(A)** Falling Numbers test (seconds) and the **(B)** spike-wetting test (sprouting score), planting dates, harvest dates, and general statistics are reported.

**Location^a^**	**Year**	**Planting Date**	**Harvest date^b^**		**Rain event precipitation**	**Rain event temperature**	**n^c^**	**t rep^d^**	**Mean ± SD^e^**	**Range (min/max)**
			**PM**	**HM**	**cm**	**°C**				
**(A)**										
Pullman	2013	–^f^	–	Aug 15, 2013	0.43	22 ± 3	459	1	379 ± 55	88/504
**Central Ferry**	**2014**	Oct 2, 2013	–	Aug 6, 2014	1.91	26 ± 5	458	2	331 ± 39	202/538
**Pullman**	**2015**	Oct 8, 2014	–	Aug 1, 2015	0.00	–	464	2	326 ± 31	187/410
**Central Ferry**	**2015**	Oct 1, 2014	–	Jul 31, 2015	3.80	31 ± 2	397	2	389 ± 55	111/537
Central Ferry	2016	Oct 13, 2015	–	Jul 14, 2016	1.80	71 ± 2	426	2	347 ± 54	154/538
**(B)**										
Pullman	2014	Oct 10, 2013	Jul 11–18, 2014	–	–	–	427	5	3.9 ± 1.9	1/10
**Central Ferry**	**2014**	Oct 2, 2013	Jun 30–Jul 8, 2014	–	–	–	230	5	4.2 ± 1.7	1/9
**Pullman**	**2015**	Oct 8, 2014	Jul 1–10, 2015	–	–	–	416	5	4.05 ± 2.2	1/10
**Central Ferry**	**2015**	Oct 1, 2014	Jun 15–25, 2015	–	–	–	275	5	5.8 ± 2.3	1/10
Pullman	2016	Oct 12, 2015	Jul 15–22, 2016	–	–	–	437	5	6.5 ± 1.9	1/10

a *Pullman, WA and Central Ferry, WA. Bold environments had both the FN and spike-wetting test conducted whereas the other environments had either the FN or spike-wetting test conducted*.

b *FN samples were harvested at harvest maturity (HM) whereas spike-wetting test samples were harvested at physiological maturity (PM)*.

c *Number (n) of accessions harvested and conducted in the FN and sprouting tests*.

d *Technical (t) replicates used for each test and environment*.

e *Mean and standard deviation (SD) were calculated; Sprouting scores from Day 5 are reported*.

f *Planting date was not recorded*.

Reduced FN was examined in field-grown material after PHS induced either by natural or artificial rain events. PHS-inducing natural rain events occurred after physiological maturity in Pullman 2013 and Central Ferry 2016. Therefore, these two environments were not used for spike-wetting tests, since the material was already sprouted. In Pullman 2013, rain occurred over 3 consecutive days with precipitation amounts of 0.38, 0.025, and 0.025 cm, and high temperatures of 22.8, 18.2, and 23.8°C, respectively (AgWeatherNet, 2016 weather.wsu.edu). Central Ferry 2016 received 1.8 cm of precipitation over 4 days with an average maximum temperature of 17°C. Artificial rain was used to induce PHS in Central Ferry 2014 and 2015 using overhead sprinkler irrigation at 2 weeks past the average physiological maturity date of the trial (precipitation = 1.91, 3.8 cm, average maximum temperature = 26 and 31°C, respectively). Approximately, 0.64 cm was applied daily over 3 days in order to induce mild sprouting. Pullman 2015 was included in the analysis as a “no event” control because there was no natural or artificial rain event after physiological maturity. Grain was harvested from plots 2–3 weeks after physiological maturity for FN tests (see below) when grain moisture was <12%. In Pullman, single plot replicates were harvested using a Wintersteiger Classic small plot combine (Wintersteiger Ag, Ried im Innkreis, Austria). In Central Ferry, one headrow was hand-harvested with a sickle per accession and machine threshed using the Vogel headrow thresher (Bill's Welding, Pullman, WA). All harvested grain was cleaned of chaff using a gravity cleaner.

### Preharvest sprouting evaluation

Spike-wetting tests were used to evaluate preharvest sprouting tolerance of field samples (Anderson et al., 1993). Intact spikes were hand-harvested from the field at physiological maturity, and allowed to dry after-ripen for the 5 days before storing at −15°C to maintain dormancy until tests were conducted (within 2–4 months). A representative set of spikes were tested for moisture content at physiological maturity, following 5 d of after-ripening, and after storage at −15°C, and found to be an average of 31, 14, and 10%, respectively. Spike-wetting tests were conducted in a greenhouse with a 16 h day/8 h night photoperiod and 22–25°C day and 16°C night temperature. Supplemental lighting was used to maintain the photoperiod with a light intensity of 300–400 μmol/m^2^/s. Spikes were misted for 6 s every minute. The rare moldy spikes were thrown out of the experiment. Sprouting scores based on the McMaster and Derera (1976) 1–10 scale were determined every 24 h for 7 days, except that the “11” value was not used. Note that no sprouting was observed until day 3 of each experiment. Since the greenhouse misting system could test a maximum of 194 genotypes at a time, each environment had to be tested over multiple weeks. Two PHS tolerant controls, “Brevor” and “Clark's Cream,” and two PHS susceptible controls, “Greer” and “Bruneau,” were included in every experiment as a check for consistency (Walker-Simmons, 1987; Tuttle et al., 2015). Spikes were arranged in a randomized order, including five technical replicates (i.e., five spikes) for each genotype.

Analyses of the spike-wetting tests were performed using sprouting scores and a sprouting index designed to give more weight to earlier than later sprouting. Sprouting index (SI) was calculated as (7 × s_day1_ + 6 × s_day2_ … + 1 × s_day7_)/(7 × n) where s is the sprouting score on each day and n is the maximum sprouting score. SI ranged from 0 to 1, where an SI of 1 indicated that the spike reached 100% highly sprouted by day 1 of misting. For the day 3, 4, 5, 6, and 7 sprouting scores and for the SI of each accession, best linear unbiased predictors (BLUPs) were calculated within each environment over the five technical replicates using the MIXED procedure in SAS/STAT v9.4 (Piepho et al., 2008). Furthermore, the week the accession was tested and the tray location in the misting system were used as covariates in the model and accessions were treated as random effects.

### Falling number evaluation

The Hagberg-Perten FN test was conducted using cleaned machine-threshed grain from Central Ferry, WA in 2014, 2015, and 2016 and from Pullman, WA in 2013 and 2015 (Table 1). FN can gradually increase during storage at higher temperatures (Ji and Baik, 2016). Grain was stored in sealed containers at −15°C to reduce problems with increasing FN. The FN test was conducted according to the ICC standard No. 107/1 (1995) and the AACC Method 56-81.03A (1999) expect that a 25 g sample was used to represent a plot or headrow rather than a 250 g sample used to represent a field. Twenty-five grams of grain was ground to meal using a Udy Cyclone Sample Mill with a 0.5 mm screen, and stored in air-tight 2 oz jars. Meal moisture content was averaged over four random samples, and applied to a subset of 48 samples. The sample weight used for the test was adjusted for moisture in order to be equivalent to 7 g of meal at 14% moisture. After 25 mL of distilled water was added to a sample, it was placed in a shaker for 5 s, then placed in a Perten Falling Number machine (Model 1600 or 1700). The FN machine determines the time needed for a stirrer to fall to the bottom of the tube after stirring and heating the samples for 60 s (minimum FN is 60 s). A lower FN is indicative of more α-amylase digestion, leading to lower gelling capacity. The FN was corrected for an altitude of 2500 ft (762 m) using FGIS Directive 9180.3 (2009). The material was examined using two technical replicates per accession with the exception of Pullman 2013, which only had one technical replicate. Each technical replicate was run on different days in 2014, within 5 min of one another in 2015, and side-by-side in 2016.

BLUPs for FN were also calculated over the artificial rain environments, the natural rain environments, and the no-rain event environment. Due to each year and environment having a different rain or no-rain event, we did not analyze BLUPs over all years or all environments (Supplementary Figure 2). An analysis of variance between accessions was performed using the MIXED procedure in SAS/STAT v9.4. Covariates were added to the analysis when relevant and included the individual who ran the test, the FN Machine used, and the seed-moisture sample subset (sets of 48 milled and tested together).

Spearman's significant rank correlations between the FN and the sprouting scores were conducted using the CORR procedure in SAS/STAT v9.4 (Supplementary Table 2). Since this is an association panel, the genotypic repeatability (*R*^2^), rather than the heritability (H^2^), was calculated using the lme4 package v1.1-13 in R (Campbell and Lipps, 1998; Bates et al., 2015). For the repeatability calculations, genotypes and covariates were considered to be random effects, whereas FN, sprouting score, or SI was used as the dependent variable.

### Genotyping

DNA was harvested and extracted as described in Froese and Carter (2016). Extracted DNA was genotyped using the Illumina Infinium iSelect 90K SNP array, and polymorphic markers were identified and curated using GenomeStudio v2011.1 (Illumina) (Wang et al., 2014). Monomorphic markers were filtered out based on the criteria of only having 0 or 1 accession with an alternate allele (out of 469). Markers with 20% or more missing data and minor allele frequency (MAF) <5% were excluded from the analysis. A consensus map consisting of SNP markers were used to align chromosome locations of polymorphic markers (Wang et al., 2014). Genetic locations of unmapped (unk for unknown) markers were cross referenced with the GrainGenes database and are reported, without reference to a cM position on a chromosome (www.graingenes.org). Missing values for markers with published locations were imputed using default parameters in BEAGLE v3.3.2 (Browning and Browning, 2016). This resulted in a total of 15,229 polymorphic markers, of which 12,681 had known locations covering all chromosomes.

### Genome-wide association study

The GAPIT R package identified three principal component sub-groups associated with market class and breeding program of origin in the mapping panel (Tang et al., 2016). Variances captured by the first three principal components (PCs) accounted for 29.6% of the total variance among the genotypes (Liu et al., 2017). In order to account for the presence of population structure, the top three PCs were fitted into the model as fixed effects (Supplementary Figure 1).

A portion of the data were analyzed using multiple statistical models, and the best statistical method selected based on how the observed *p*-values exceeded the null expectation on the Q-Q plot from GAPIT and FarmCPU (Lipka et al., 2012; Liu et al., 2016; Tang et al., 2016). Using the Pullman 2014 FN and sprouting score phenotypic data, a general linear model (GLM), mixed linear model (MLM), compressed mixed linear model (CMLM), SUPER model, and FarmCPU model were compared (Zhang et al., 2010; Wang et al., 2014; Liu et al., 2016; Supplementary Figure 3). The results indicated that the FarmCPU model performed better than the other models. All subsequent genome-wide association analyses used only the FarmCPU model.

FarmCPU default parameters were used except that the “optimum” bin method with default range and interval parameters was used instead of the “static” method. The FN trait least squares means (LSMeans) and the sprouting score BLUPs were used as dependent variables in this GWAS (Supplementary Figure 3). Markers were identified as significantly associated with the trait after a 1% Bonferroni multiple test correction (*p* < 2.85E-07; −log_10_(*p*) > 6.55).

The proportion of explained phenotypic variance was calculated as follows:

$$r^{2}=\frac{\sum_{i=1}^{n}{\left({\hat{y}}_{i}-\hat{y}\right)}^{2}}{\sum_{i=1}^{n}{\left(y_{i}-y\right)}^{2}}$$

where *y*_*i*_ is observed phenotype value, $\hat{y_{i}}$ is the estimated phenotype value from a multiple linear regression model that was fitted to all significant SNPs as an independent variable with fixed effect.

Linkage disequilibrium (LD) was calculated using JMP software v6.0 (SAS, Cary, NC). LD of significant markers were used to estimate boundaries of potential quantitative trait loci (QTL) using the criteria of LD (*R*^2^ > 0.2), chromosome location (cM) based on Wang et al. (2014), correlation between markers, and marker-trait information among the significant markers. For each designated QTL, the marker with the strongest association with either FN or sprouting scores was reported. Criteria for a strong association include: (a) phenotypic variation explained by the marker (*r*^2^); (b) allelic effect; and (c) marker *p* value.

Tolerant nucleotides of each significant marker were used to determine the pyramiding effect of PHS tolerant loci. A linear model regression was applied to the phenotypic estimates and number of favorable loci per accession. “Favorable” loci nucleotides were those that lowered the sprouting score or increased the FN. Pearson's correlation coefficients were calculated between the trait and number of favorable loci using the “cor” function in R v.3.2.5.

### Comparison of QTN locations with previously reported PHS genes and QTL

A comparison of identified QTN with previous studies was performed using the integrated map of Maccaferri et al. (2015) that includes SSR markers, 9k SNP markers, 90k SNP markers, Synthetic × Opata DH GBS markers, and the Diversity Array Technology markers (Supplementary Table 3; Akbari et al., 2006; Cavanagh et al., 2013; Saintenac et al., 2013; Wang et al., 2014). Maccaferri et al. (2015) converted distances in cM into relative % length distances by dividing them by total chromosome length. The approximate relative positions of QTN were estimated based on known marker positions. Note that the LD of QTL from other studies were not recalculated.

## Results

### Environmental response of PHS-related traits in soft white wheat

**Figure 1:**
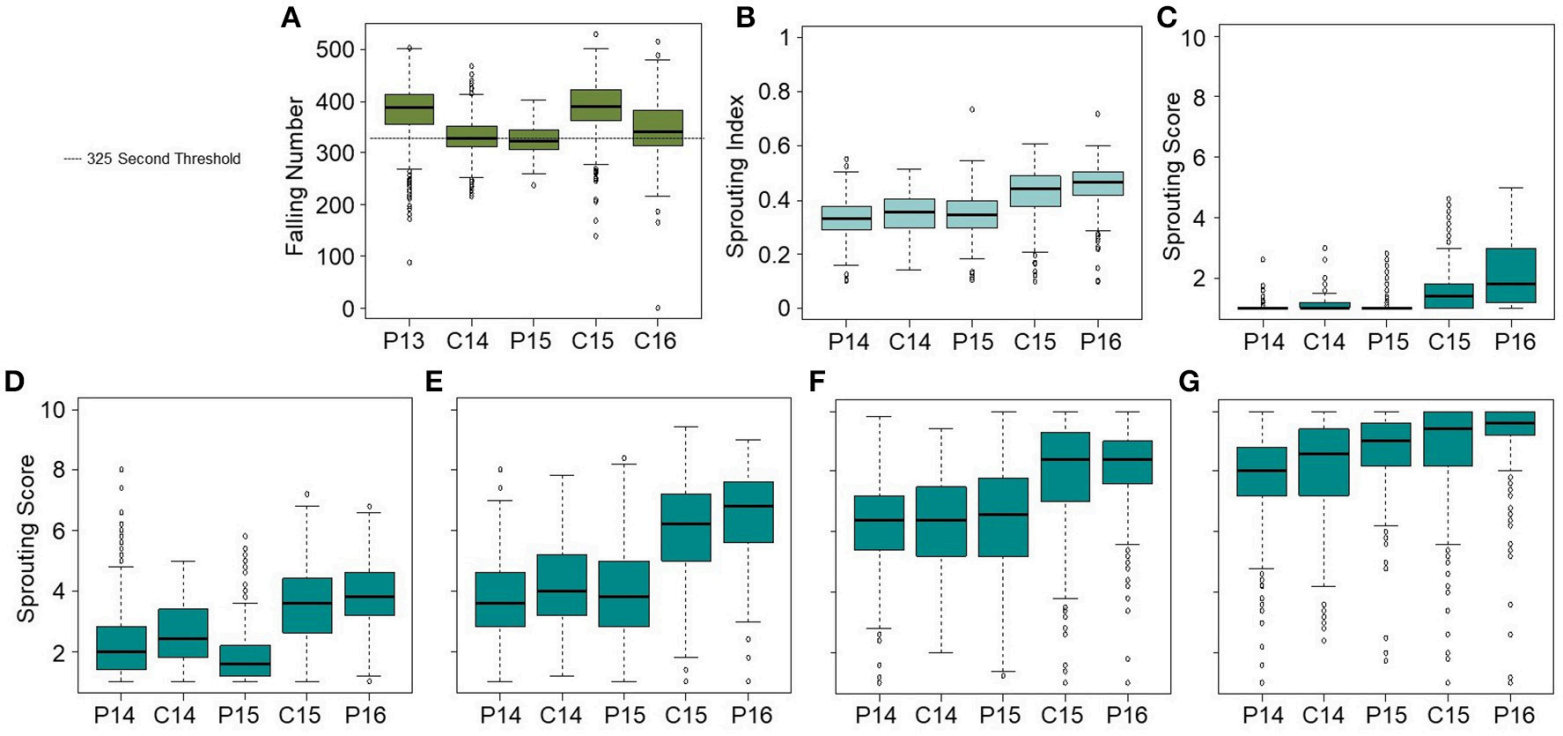
FN and PHS distributions. **(A)** FN distributions across environments including the natural rain events in Pullman 2013 (P13) and Central Ferry 2016 (C16), an artificial rain event in Central Ferry 2014 (C14) and 2015 (C15), and an environment without rain in Pullman 2015 (P15). Spike-wetting tests were performed for Pullman 2014 (P14) as well as C14, P15, C15, and Pullman 2016 (P16). **(B)** Sprouting index was calculated over all 7 days of misting. Sprouting scores after **(C)** 3, **(D)** 4, **(E)** 5, **(F)** 6, and **(G)** 7 days of misting were based on a 1-10 scale.

A panel of 469 soft white wheat accessions was evaluated for PHS tolerance based on spike-wetting tests and FN following rain in the field. FN was examined in five environments over 4 years and at two locations. The mean FN ranged from 326 to 389 s for all environments (Table 1). Increasing α-amylase activity tends to correlate with decreasing FN below 325 s (Perten, 1964; Yu et al., 2015). All environments had multiple accessions below the 325 s threshold (ranging from 36 to 234 accessions; Figure 1; Supplementary Figure 2). Natural sprout-inducing rain events occurred in Pullman 2013 and Central Ferry 2016. Artificial rain was applied to induce sprouting in Central Ferry in 2014 and 2015. Pullman 2013, and Central Ferry 2015 and 2016 had wide variation for FN, but Central Ferry 2015 had very few accessions below 325 s. The Pullman 2015 data was included in the analysis as a “no-rain-event” control in an attempt to differentiate PHS-induced differences in FN from differences in FN due to starch or protein composition (AgWeatherNet, 2016 weather.wsu.edu). Pullman 2015 had 234 accessions below 325 s but had lower variation than other environments. This is likely due to poor grain filling since that season had unusually high summer temperatures. QTN mapped based on FN data will be referred to as *QFN.wsu*.

In spike-wetting tests, environments had similar effects on results regardless of whether we examined sprouting scores or the sprouting index. For example, Central Ferry 2015 and Pullman 2016 consistently showed the highest level of sprouting (Figure 1; Table 1). BLUP analysis was performed in order to reduce environmental variation in sprouting scores prior to mapping (Supplementary Figure 2). BLUPs calculated over all environments were highly correlated over days of sprouting and with the sprouting index (SI) (Supplementary Table 2A). Based on the range of values, we expected to have enough variation to map QTN associated with PHS tolerance due to reduced germination or slower post-germinative growth. For ease of communication, QTN identified based on sprouting scores or SI will be referred to collectively as PHS loci or *QPHS.wsu*.

### PHS trait correlations

Correlations were used to examine whether genotypic differences in FN or sprouting scores were consistent between environments, and to examine whether FN and sprouting scores were related. The FN values showed a weak but significant positive correlation between environments, ranging from 0.23 to 0.46 (*p* < 0.001; Table 2). Sprouting scores were positively correlated between environments, but not always significantly (Supplementary Tables 2B–G). Day 3 sprouting scores showed the least significant correlation between environments, whereas day 5 and day 6 sprouting scores showed significant positive correlations between environments. SI had the strongest and most significant correlation between environments. The genotypic repeatability of FN (*R*^2^ = 0.50) was greater than sprouting scores (*R*^2^ < 0.31), especially when covariates like operator, machine, subset, and replicate, were taken into account (Table 3). When repeatability was calculated on a line mean basis, both traits had similar repeatability in our experiments (FN *R*^2^ = 0.67; SI *R*^2^ = 0.70). The genotypic repeatability of sprouting was highest when spikes were misted longer or when all measurements were integrated in the SI (Table 3). Because seed germination induces α-amylase, which in turn lowers FN, we expected higher sprouting scores to negatively correlate with lower FN, but this was not the case (Table 2). BLUPs for SI generated over all days scored showed the strongest and most significant negative correlation with FN in years when natural sprouting events occurred (Table 2B). In Central Ferry 2015, the FN did not correlate to the sprouting scores and SI. Overall, 4 days of misting had the highest correlation across all FN environments.

**Table 2:** Rank correlation coefficients for **(A)** FN LSMeans across environments and **(B)** PHS score BLUPs compared to FN environments.

**(A)**	**FN:**	**P13^a^**	**C14**	**P15**	**C15**		
FN	C14	0.29^**^					
	P15	0.23^**^	0.42^**^				
	C15	0.23^**^	0.29^**^	0.29^**^			
	C16	0.33^**^	0.46^**^	0.30^**^	0.34^**^		
**(B)**	**PHS:**	**3 days**	**4 days**	**5 days**	**6 days**	**7 days**	**SI**
FN	P13	−0.16^**^	−0.24^**^	−0.17^**^	−0.18^**^	−0.20^**^	−0.21^**^
	C14	−0.07	−0.09^*^	−0.06	−0.09	−0.10^*^	−0.10^*^
	P15	−0.07	−0.13^*^	−0.12^*^	−0.12^*^	−0.17^**^	−0.15^**^
	C15	−0.09	−0.04	0.00	0.01	0.00	−0.01
	C16	−0.17^**^	−0.19^**^	−0.18^**^	−0.17^**^	−0.17^**^	−0.19^**^

* Represents a p-value ≤ 0.05 and

** *represents a p-value ≤ 0.001*.

a *Environments Pullman (P) and Central Ferry (C)*.

**Table 3:** Genotypic repeatability (*R*^2^) of FN, spouting scores, and SI across all environments.

**Trait**	**Simple *R*^2^^a^**	**Covariate *R*^2^^b^**	**Line mean basis *R*^2^^c^**
FN	0.197	0.500	0.667
PHS d3 ^d^	0.109	0.145	0.459
PHS d4	0.163	0.240	0.612
PHS d5	0.151	0.214	0.577
PHS d6	0.229	0.276	0.656
PHS d7	0.218	0.230	0.599
SI	0.228	0.315	0.697

a *Genetic and environmental variances were calculated using a simple y ~ x model with x (genotypes) as a fixed effect and repeatability R ^2^ = V_g_ / (V_g_ –V_e_) was calculated*.

b *Genetic and environmental variances were calculated using a simple y ~ x + covariates model with x (genotypes) and covariates (time, machine, operator, etc.) as a fixed effect*.

c *Repeatability R ^2^ = V_g_/(V_g_ –(V_e_ /n)) was expressed by a line mean basis by dividing the environmental variance (V_e_, residuals) with the number of technical reps (n; FN = 2 and spike-wetting test = 5)*.

d *Sprouting score (PHS) on days (d) 3 through 7*.

### Association analysis for falling numbers

GWAS for FN was performed using FarmCPU within each environment because environments varied drastically. All environments fit the FarmCPU association mapping model (Supplementary Figure 4). However, Central Ferry 2015 showed no significant marker-trait associations, regardless of which model was used for GWAS (Supplementary Figure 3D). Nine significant QTN associated with FN were mapped on chromosomes 4A, 5A, 5D, 7A, and 7B (Table 4). The three QTN identified without rain in Pullman 2015 likely represent grain characteristics that are independent of PHS. The remaining QTN were identified in natural rain events. The QTN detected on chromosomes 6D (–log_10_(*p*) = 5.88) and 7A (–log_10_(*p*) = 6. 17) in Central Ferry 2014 were just below the stringent threshold for significance (–log_10_(*p*) = 6. 55). *QFN.wsu-7A.1* and *QFN.wsu-7B.2* had the largest effects, increasing FN by 26 and 27 s, respectively. *QFN.wsu-7A.2* had the highest significance (–log_10_(*p*) = 12.36) and had an 8 s effect. In order to reduce the effect of loci unrelated to PHS tolerance on the GWAS, the data were re-analyzed with 400 s set as the maximum possible FN. When this was done, none of the nine *QFN.wsu* were detected and two unique significant QTN, *QFN.wsu-6A* and *QFN.wsu-7A.3* were identified in Central Ferry 2016 (Supplementary Table 4). These two QTN had large effects, increasing FN by 17 and 13 s, respectively, but only explained 1% of the phenotypic variation. A GWAS was also conducted for heading date from Pullman 2014 and there were no heading date QTN discovered that overlapped with QTNs for either FN or PHS (Supplementary Table 5).

**Table 4:** Loci significantly associated with Falling Numbers (FN), early preharvest sprouting (PHS) scores (days 3–4), 5 days of misting, late PHS scores (days 6–7), and PHS sprouting index (SI).

**QTL^a^**	**Marker**	**Chr^b^**	**cM^b^**	**–log10(*p*)**	**maf**	**Effect^c^**	***r^2^***	**Environment**	**Favorable Allele^d^**
QFN.wsu-4A^*^	IWB1884	4A	152	6.63	0.48	10.28	0.00	C16 FN	**A**/C
QFN.wsu-5A.1^*^	IWB60191	5A	23	7.27	0.27	7.53	0.00	P15 FN	**A**/G
*QFN.wsu-5A.2*	IWB9800	5A	141	7.77	0.20	7.43	0.00	P15 FN	**A**/G
*QFN.wsu-5D*	IWB36060	5D	202	6.11	0.35	11.70	0.08	P13 FN	A/**C**
*QFN.wsu-7A.1*	IWB22966	7A	35	8.34	0.06	26.09	000	P13 FN	A/**G**
*QFN.wsu-7A.2*	IWA334	7A	126	12.36	0.41	7.99	0.01	P15 FN	A/**C**
*QFN.wsu-7B.1*	IWB39063	7B	162	7.91	0.48	10.88	0.01	C16 FN	A/**G**
*QFN.wsu-7B.2*	IWB75387	7B	–	6.15	0.09	27.35	0.00	P13 FN	A/**C**
*QFN.wsu-unk*	IWB37658	unk	–	6.82	0.09	15.49	0.00	C16 FN	T/**C**
*QPHS.wsu-1A.1*	IWB2320	1A	82	6.73	0.15	−0.04	0.00	P14 d3	**T**/C
**QPHS.wsu-1A.2**	IWB6759	1A	155	11.70	0.47	−0.31	0.15	P14 d3	**A**/G
	IWB77968	1A	155	12.72	0.47	−0.02	0.10	P14 d3	**A**/G
**QPHS.wsu-1B.2**	IWB64868	1B	135	9.00	0.15	−0.40	0.17	C14 d4	**A**/G
	IWB31676	1B	137	8.18	0.08	−0.37	0.00	P16 d3	**A**/G
*QPHS.wsu-2A.1*	IWB42693	2A	25	6.87	0.22	−0.16	0.02	C15 d3	**T**/G
*QPHS.wsu-2D*	IWB7652	2D	52	10.03	0.37	−0.46	0.00	C14 d4	T/**C**
	IWA8544	2D	50	8.73	0.46	−0.28	0.01	P16 d3	**A**/G
	IWA8544	2D	50	9.12	0.46	−0.32	0.07	P16 d4	**A**/G
*QPHS.wsu-3A.2*	IWB50719	3A	68	6.71	0.14	−0.29	0.04	C14 d4	A/**G**
*QPHS.wsu-4A.1*	IWA7535	4A	58	8.57	0.05	−0.07	0.03	P14 d3	A/**G**
*QPHS.wsu-4B.2*	IWB21707	4B	75	8.93	0.10	−0.42	0.07	P14 d4	A/**G**
QPHS.wsu-4B.3^*^	IWB22055	4B	101	6.57	0.08	−0.37	0.00	P16 d3	A/**G**
*QPHS.wsu-5B.1*	IWB31067	5B	26	8.75	0.08	−0.06	0.01	P14 d3	T/**G**
*QPHS.wsu-7B.1*	IWB54418	7B	3	7.46	0.03	−0.26	0.01	P16 d3	A/**G**
QPHS.wsu-1B.1^*^	IWB22868	1B	31	7.88	0.18	−0.30	0.01	P14 d5	**T**/C
*QPHS.wsu-2D*	IWB46396	2D	54	9.63	0.39	−0.49	0.02	C14 d5	**A**/G
*QPHS.wsu-3B.2*	IWA6185	3B	62	6.55	0.44	−0.23	0.01	P14 d5	A/**G**
*QPHS.wsu-4A.2*	IWB54609	4A	66	7.30	0.17	−0.35	0.01	P16 d5	**A**/G
QPHS.wsu-5A.2^*^	IWB10250	5A	70	9.13	0.32	−0.44	0.03	P15 d5	**T**/C
*QPHS.wsu-5B.3*	IWB73511	5B	129	6.73	0.30	−0.28	0.01	P16 d5	A/**G**
QPHS.wsu-6B^*^	IWA1838	6B	65	10.53	0.07	−0.29	0.05	P14 d5	**A**/G
QPHS.wsu-7B.2^*^	IWB7099	7B	133	9.00	0.00	−0.34	0.00	C14 d5	A/**G**
QPHS.wsu-1D^*^	IWB71680	1D	163	7.61	0.06	−0.60	0.09	P14, P16 d6	**A**/G
QPHS.wsu-2A.2^*^	IWB17580	2A	53	9.02	0.07	−0.69	0.02	C15 d7	T/**C**
*QPHS.wsu-2A.3*	IWB79387	2A	–	6.88	0.01	−0.31	0.00	C14 d6	A/**G**
**QPHS.wsu-2D**	IWB7652	2D	52	12.69	0.37	−0.85	0.12	C14 d6, d7	T/**C**
*QPHS.wsu-3A.1*	IWB32631	3A	15	6.63	0.26	−0.31	0.02	C14 d7	A/**G**
QPHS.wsu-3B.1^*^	IWB6430	3B	11	8.92	0.08	−0.38	0.01	P14 d7	T/**C**
*QPHS.wsu-3B.3*	IWB9902	3B	–	7.76	0.07	−0.59	0.06	P14 d7	**T**/C
*QPHS.wsu-4A.2*	IWB46089	4A	73	6.83	0.16	−0.33	0.04	P16 d6	A/**G**
*QPHS.wsu-4A.3*	IWB1389	4A	151	7.51	0.23	−0.45	0.02	C14 d7	**T**/G
*QPHS.wsu-4B.1*	IWB72936	4B	60	7.92	0.25	−0.46	0.02	C14 d7	**A**/G
*QPHS.wsu-4B.2*	IWA1382	4B	73	8.04	0.06	−0.53	0.00	P15 d7	A/**G**
*QPHS.wsu-5A.2*	IWB60303	5A	70	7.56	0.34	−0.39	0.01	C15 d7	A/**G**
QPHS.wsu-5A.3^*^	IWB6049	5A	84	9.92	0.19	−0.31	0.00	P16 d6	**A**/G
*QPHS.wsu-6A*	IWB6726	6A	77	7.52	0.07	−0.48	0.03	P14 d7	**T**/G
*QPHS.wsu-6B*	IWB76583	6B	65	9.76	0.05	−0.33	0.03	P14 d6	**A**/G
QPHS.wsu-6D^*^	IWB49280	6D	153	7.17	0.03	−0.39	0.00	P15 d7	**A**/G
QPHS.wsu-7A^*^	IWB51129	7A	152	6.64	0.00	−0.27	0.06	P16 d6	A/**G**
*QPHS.wsu-7B.3*	IWB10815	7B	171	10.62	0.05	−0.41	0.00	P14 d7	T/**C**
QPHS.wsu-1D^*^	IWB71680	1D	163	7.22	0.06	−0.03	0.10	P16 SI	**A**/G
*QPHS.wsu-2B*	IWB30853	2B	87	7.59	0.21	−0.02	0.00	C14 SI	A/**G**
*QPHS.wsu-2D*	IWB46396	2D	54	11.97	0.39	−0.03	0.07	C14 SI	**A**/G
*QPHS.wsu-3B.3*	IWB9902	3B	–	7.31	0.07	−0.03	0.00	P14 SI	**T**/C
***QPHS.wsu-5A.1***	IWB10998	5A	53	8.70	0.41	−0.02	0.25	C14 SI	T/**C**
*QPHS.wsu-6B*	IWB57747	6B	64	6.85	0.07	−0.02	0.07	P16 SI	**A**/G
	IWB76583	6B	65	6.57	0.02	−0.01	0.00	P14 SI	**A**/G
QPHS.wsu-7B.2^*^	IWB7099	7B	133	8.63	0.00	−0.02	0.01	C14 SI	A/**G**
	IWB7099	7B	133	7.58	0.01	−0.02	0.00	P16 SI	A/**G**

a QTL in bold explained 10% (r^2^ > 0.1) or more of the phenotypic variation. QTL underlined were significant in 2 environments. Loci more than 10 cM away from previously published QTL were considered to be novel and are indicated with an^*^.

b *Chromosome and position according to Wang et al. (2014). Positions are not reported if the location was identified on the GrainGenes database*.

c *The allelic effect is shown in FN seconds or sprouting score BLUPs*.

d *The significant allele is favorable (in bold) if it decreases sprouting scores in the spike-wetting tests or increases Falling Numbers*.

### Association analysis for sprouting score and sprouting index

**Figure 2:**
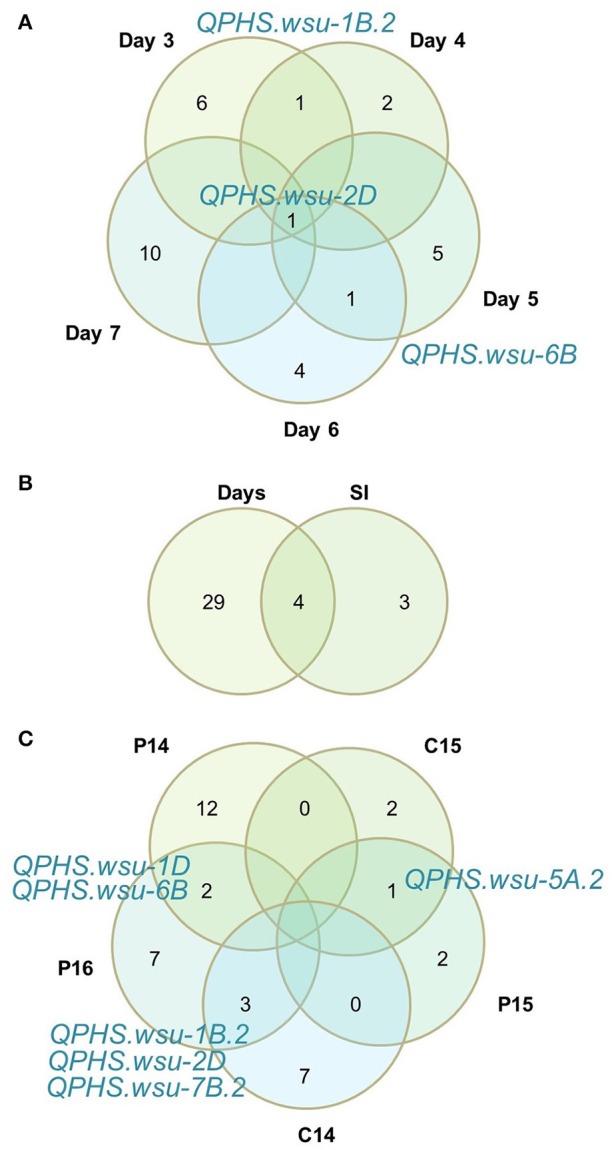
Venn diagrams comparing sprouting score QTN across **(A)** days misted, **(B)** all days misted and sprouting index, and **(C)** the five environments. Shared QTN are printed in blue.

For the spike-wetting tests, 34 significant *QPHS.wsu* were detected based on sprouting scores or on SI (Table 4). There were 12 *QPHS.wsu* associated with early germination and root emergence (3–4 days of misting) (Figure 1; Table 4; McMaster and Derera, 1976). Seven *QPHS.wsu* were identified after 5 days of misting, only one of which was also seen with 3–4 days of misting. There were 16 significant *QPHS.wsu* associated with coleoptile emergence and elongation at 6–7 days of misting, 13 of which were unique to 6–7 days of misting. There were 3 additional *QPHS.wsu* uniquely detected by SI (Figure 2B). An association at the *QPHS.wsu-2D* locus was detected over all days of scoring, suggesting that it is not unique to any one sprouting stage (Figure 2A). The *QPHS.wsu-1B.2* and *QPHS.wsu-6B* were detected on two of the scoring days. Of the 34 total QTN found in the spike-wetting tests, only 6 *QPHS.wsu* on chromosomes 1B, 1D, 2D, 5A, 6B, and 7B were significant in two environments (Figure 2C). No *QPHS.wsu* were detected in more than 2 environments. Five *QPHS.wsu* on chromosomes 1A, 1B, 1D, 2D, and 5A explained more than 10% of the phenotypic variation. In Central Ferry 2015, only sprouting scores on days 3 and 7 fit the expected −log(*p*), whereas days 4, 5, 6, and SI did not (Supplementary Figure 4). Therefore, very few significant *QPHS.wsu* were identified in this environment.

In order to reduce the effect of post-germinative growth versus germination *per se*, the GWAS was repeated with the maximum sprouting score set at 5 and referred to as *QPHSg.wsu* (Supplementary Table 4). Out of 46 QTN detected, 32 QTN were unique to this analysis and 21 were no longer significant (Supplementary Figure 5). The major QTN, *QPHS.wsu-1D, QPHS.wsu-2D, QPHS.wsu-5A.2*, and *QPHS.wsu-7B.2* were also detected in this germination-based analysis. The *QPHSg.wsu-2D* locus was highly significant in this GWAS (7.64 < –log_10_(*p*) < 37.13), and had strong effects (0.02 to 0.72).

### Association analysis without using the club wheat breeding program as a covariate

**Figure 3:**
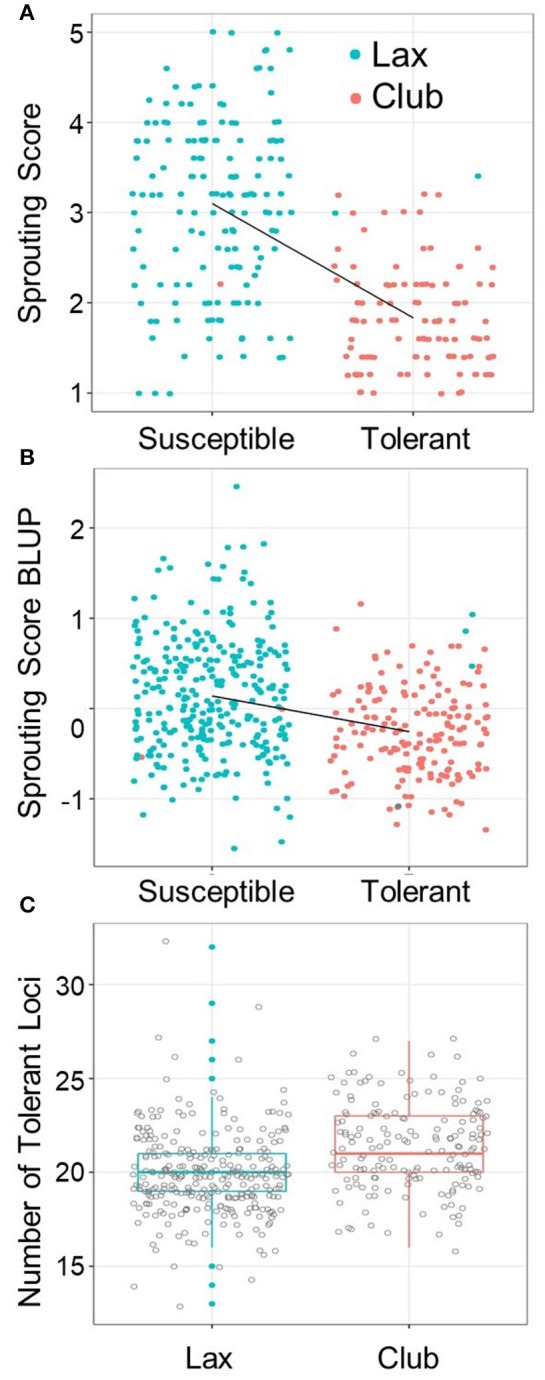
*QPHS.wsu-2D* in club versus lax mapping lines. Scatter plot of sprouting score **(A)** raw means over the environment, Central Ferry 2014, that *QPHS.wsu-2D* had the strongest effect and **(B)** Sprouting score BLUPs across all five environments after 4 days of misting versus the presence of the *QPHS.wsu-2D* susceptible (S) or tolerant (T) allele. Club genotypes are in red and lax in blue. **(C)** Total number of favorable loci are compared to club versus lax spike morphology.

Based on the fact that the strong *QPHS.wsu-2D* locus was near the *C* locus region for compact spike morphology, we examined whether the club wheat breeding program contributed more PHS tolerance to the GWAS than the lax wheat breeding programs (Figure 3). In Central Ferry 2014, when the tolerant and susceptible loci from the strongest marker within *QPHS.wsu-2D* were compared, 98% of the tolerant loci were club (Figure 3A). When the BLUPs were compared across all environments, the same trend was observed (Figure 3B). In fact, the club wheat breeding program generally contributed more PHS tolerance in the GWAS than the other programs (Figure 3C). Thus, it is possible that using the principle components as a covariate in the GWAS may artifactually remove some of the PHS loci contributed by the club wheat breeding program. To test this hypothesis, the GWAS was repeated without incorporating the principle components into the model (Supplementary Table 6). The *QPHS.wsu-2D* QTN became stronger in this analysis, increasing to a –log_10_(*p*) of up to 30.03 and an effect of 1.08. An additional 32 *QPHSnPC.wsu* and 2 *QFNnPC.wsu* were detected, whereas 15 *QPHS.wsu* and 2 *QFN.wsu* were found in common (Supplementary Figure 5).

### Pyramiding effects of FN and PHS QTN

**Figure 4:**
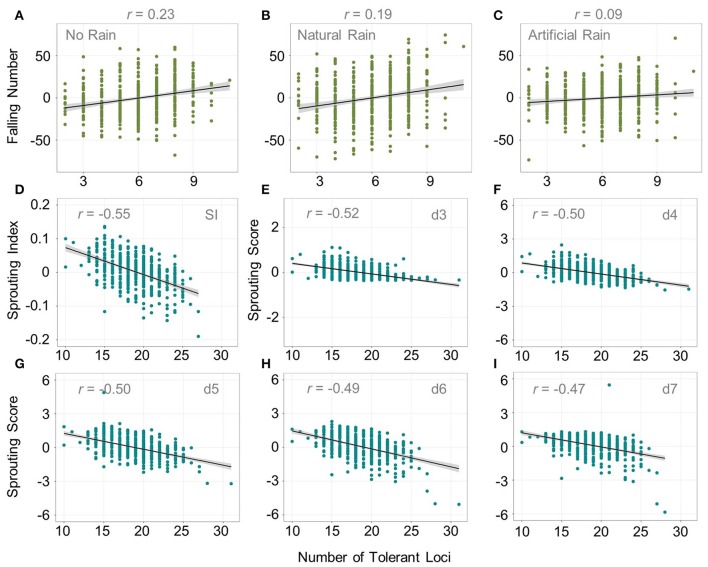
The effect of pyramiding multiple *QFN.wsu* and *QPHS.wsu* loci. Scatter plots of the number of favorable *QFN.wsu* loci versus FN BLUPs across: **(A)** in the absence of rain, **(B)** both natural rain environments combined, and **(C)** both artificial rain environments combined. Scatter plots of the number of favorable *QPHS.wsu* loci versus BLUPs calculated across all environments for **(D)** sprouting index, and sprouting scores on days **(E)** 3, **(F)** 4, **(G)** 5, **(H)** 6, and **(I)** 7 of misting. *r* is the Pearson correlation coefficient between the trait and number of tolerant loci.

Next, we examined whether an increasing number of favorable QTN were associated with increasing FN or PHS tolerance. The number of favorable *QFN.wsu* loci within accessions ranged from 2 to 9 (Figures 4A–C). An increasing number of *QFN.wsu* loci was only weakly correlated to higher FN. The correlation was actually stronger when there was no rain event than when there was a natural or artificial rain event (*r* = 0.23, 0.19, 0.09, respectively). The number of *QPHS.wsu* loci varied more widely within the accessions (8 to 26), making it easier to assess the effects of pyramiding multiple tolerance loci (Figures 4D–I). An increasing number of tolerance loci was negatively correlated with sprouting scores ranging from *r* = −0.47 to *r* = −0.55 (Supplementary Figure 6). Thus, having more *QPHS.wsu* loci was associated with more PHS tolerance. Tolerant FN loci only slightly correlated with the increasing sprouting scores, and vice versa (*r* < 0.20; Supplementary Figure 6).

Within the mapping panel, there was only one accession, “A00154,” with all 9 *QFN.wsu* that had an average FN of 461 s across natural rain events. The accession “6J020288-1” had the highest number of favorable alleles, including 26 out of 34 *QPHS.wsu* and 6 of 9 *QFN.wsu*. This was reflected in the phenotype since 6J020288-1 had an average FN of 380 s across natural rain events and a sprouting score of 1 after 5 days of misting from all environments. In contrast, the accession “J950409-10-2” had only 9 out of 34 *QPHS.wsu* and 5 of 9 *QFN.wsu*, associated with an average FN of 272 s and a high sprouting score of 7 after 5 days of misting. Interestingly, “Lewjain” had only 8 of the 34 *QPHS.wsu* and 5 of the 9 *QFN.wsu* but had an average FN of 401 s and a sprouting score of 4 after 5 days. Thus, these QTN do not always behave in an additive fashion, suggesting that there are epistatic effects. For example, some of the few favorable QTN in Lewjain may have a stronger effect on the phenotype than the unfavorable alleles.

### Comparative mapping for PHS

**Figure 5:**
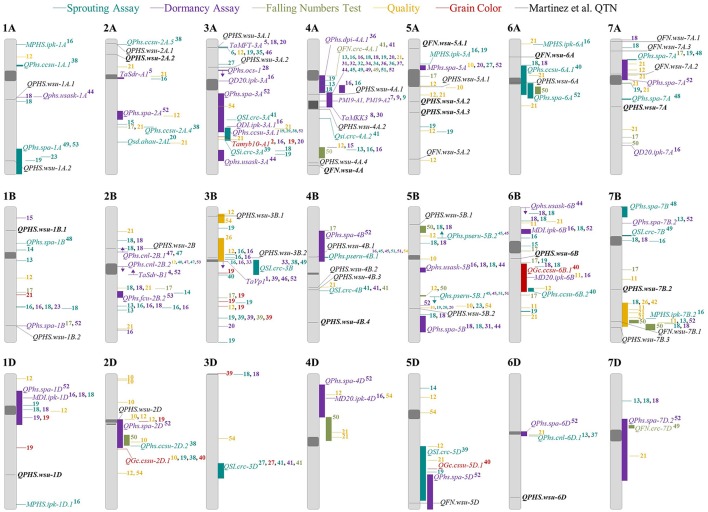
Chromosome positions of PHS-related loci. Comparative mapping of quantitative trait nucleotides identified in this study (black), and previously published quantitative trait loci (QTL) for PHS tolerance (blue), dormancy (purple), Falling Number (green), grain color (red), and quality (orange). Potentially novel *QPHS.wsu* or *QFN.wsu* QTN are in bold. Published QTL were aligned to the Maccaferri et al. (2015) comparative map using the flanking markers or significant associated markers. Chromosomes are presented as a standardized relative length. An arrow along a QTL indicates the QTL direction when only one flanking marker was found in the comparative map. The centromeric region is a dark gray oval. ID numbers to the right of the QTL correspond to the references found in Supplementary Table 3. Illumina 9 or 90 k SNP markers were not reported on chromosome 3D, resulting in an incomplete map.

The location of QTN for FN and sprouting scores were compared to locations of PHS-related loci identified in 54 previous studies (Figure 5). This was done using the comparative map of commonly used wheat markers created by Maccaferri et al. (2015). The studies used in the comparison are shown in Supplementary Table 3. Mapped traits that are related to FN and end-use quality included FN, starch content, protein content, and α-amylase activity. Mapped traits related to sprouting and dormancy included germination assays, kernel color, and sprouting scores from spike-wetting tests. There is currently no experimental standard for the spike-wetting test, and methods vary from misting of intact spike to spike immersion to the use of wet sand (McMaster and Derera, 1976; Paterson et al., 1989; Anderson et al., 1993; Humphreys and Noll, 2002; Rehman Arif et al., 2012). Even within each method, the number of days of after-ripening, the duration of spikes wetting, and the day scored vary between studies. Only a few studies score PHS after 4–6 days of misting (Anderson et al., 1993; Munkvold et al., 2009; Kulwal et al., 2012; Somyong et al., 2014). This inconsistency across studies led us to ask whether or not our assay could map previously published cloned genes and QTL. Nineteen of the 34 sprouting QTN detected in this study co-localized with known major PHS QTL and cloned genes such as *TaMFT* on chromosome 3A (Figure 5; Nakamura et al., 2011). Interestingly, 14 of the 34 sprouting QTN were identified near known FN or quality QTL. Three of 9 FN QTN were identified near known FN and quality QTL, whereas 4 of 9 FN QTN were identified near dormancy or PHS QTL. Of these FN QTN, 2 co-localized with both PHS and FN/quality QTL. A total of 2 QTN for FN and 10 QTN for sprouting score appeared to be unique to this study (marked with a star in Table 4).

## Discussion

This genome-wide association study was, to our knowledge, the first to map preharvest sprouting loci based both on sprouting scores from the spike-wetting test (*QPHS.wsu*) and FN (*QFN.wsu*). FN is an important and complex trait that determines the value of the grain in the wheat industry. While there were 34 significant *QPHS.wsu* loci across the five different sprouting time points and sprouting index, only nine significant *QFN.wsu* loci were identified. We expected FN and sprouting scores to identify some of the same QTN because the α-amylase expression that lowers FN is a consequence of germination. However, none of the identified significant FN and PHS loci were linked (Table 4). While *QFN.wsu-4A* and *QPHS.wsu-4A.3* appeared to be close to one another (1 cM apart), they were not in the same linkage group (*p* between 0.05 and 0.01), and had only a −0.13 correlation to one another. Moreover, we failed to find a strong correlation between FN and sprouting scores. This result contrasts with previous studies showing a correlation of up to −0.8 between spike-wetting tests and FN in Canadian and Chilean breeding lines (Rasul et al., 2009; Jiménez et al., 2016).

The lack of a strong correlation between FN and sprouting score in our study has multiple likely causes: our association panel was larger and sampled more variability for FN related traits; our environments were more variable; or the causes of low FN were not solely due to PHS. Eastern Washington is a semi-arid environment where grain is planted deeply to reach moisture and selection for emergence may have led to early and strong induction of α-amylase to fuel seedling growth. This, in turn, may have resulted in lower FN than would be expected for a given sprouting score. Future work may investigate this by determining if a propensity for low FN is associated with earlier expression of α-amylase during germination. The *QFN.wsu* loci identified, however, did appear to be related to preharvest sprouting because they co-localized with PHS-related loci identified in other studies. Thus, while sprouting scores were not predictive of FN in this study, both traits appeared to be useful for identifying PHS-related loci.

### Comparison to previously published PHS QTL and genes

Based on comparative mapping, 12 potentially novel PHS tolerance loci were identified (Figure 5). With the exception of *QPHS.wsu-4B.3*, the QTN identified during early sprouting (3–4 days misted) were near published QTL (Table 4). In contrast, six novel loci were identified during late sprouting (6–7 days misted). Previous PHS mapping studies mostly used spike-wetting test data collected after 4–6 days of misting (Anderson et al., 1993; Munkvold et al., 2009; Kulwal et al., 2012; Somyong et al., 2014).

Multiple *QFN.wsu* were located near published QTL or cloned genes governing PHS-related traits (Figure 5; Supplementary Table 3). Three of the six *QFN.wsu* identified in the presence of natural rain were near known preharvest sprouting and dormancy QTL (Fofana et al., 2009; Kumar et al., 2009, 2015; Kulwal et al., 2012; Albrecht et al., 2015). Previous work has shown FN samples after a rainfall negatively correlate with dormancy which may be another explanation as to why we see FN QTN near dormancy QTL (Biddulph et al., 2008). *QFN.wsu-7A.1* had the largest effect on FN, and was near PHS and dormancy QTN found in a European winter wheat GWAS (Albrecht et al., 2015). *QFN.wsu-7B.1* localized in a region containing known QTL mapped based on spike-wetting tests, spike immersion, dormancy, and FN (Kulwal et al., 2012; Mohler et al., 2014; Albrecht et al., 2015; Kumar et al., 2015). Given that these QTN were detected after a natural rainfall event, it is curious that they were not detected in any of our spike-wetting test environments. This suggests that either these *QFN.wsu* loci resulted in higher FN due to PHS tolerance, or that the co-localization of these FN QTN with sprouting QTLs was a coincidence. FN is also controlled by grain starch characteristics. Consistent with this, the *QFN.wsu-5A.2* and *QFN.wsu-7A.2* loci identified in the absence of rain were located near starch content and starch granule size QTL (Reif et al., 2011; Li et al., 2017).

Many *QPHS.wsu* were found near published QTL or cloned genes associated with seed dormancy and PHS tolerance (Figure 5). *QPHS.wsu-2A.1* was located near a preharvest sprouting QTL, *QPhs.ccsu-2A.5*, found in the dormant accession “SPR8198” (Mohan et al., 2009). *QPHS.wsu-2B* was located near the locus providing dormancy and PHS tolerance in the soft white wheat cultivar “Cayuga” and in a European winter wheat QTL (Somyong et al., 2014; Albrecht et al., 2015). The *QPHS.wsu-2D* QTN had the strongest effect, and co-localized with the *QPhs.spa-2D* locus identified in Canadian wheat (Kumar et al., 2015). *QPHS.wsu-3A.1* was within 1 cM of the major dormancy and PHS tolerance gene *MOTHER OF FT AND TFL1* (*TaMFT*), identified by map-based cloning in both Japanese and U.S. wheat (Nakamura et al., 2011; Liu et al., 2013). *QPHS.wsu-4A.1* and *QPHS.wsu-4A.2* are within the *Phs-A1* region associated with dormancy and PHS tolerance in mapping studies world-wide (Mares and Mrva, 2014; Barrero et al., 2015; Shorinola et al., 2016, 2017; Torada et al., 2016). A polymorphism in the *MITOGEN-ACTIVATED PROTEIN KINASE KINASE 3* (*TaMKK3-A*) gene likely accounts for the seed dormancy providing PHS tolerance on chromosome 4A. Future work will need to examine if the PHS tolerance loci mapped in the current study are associated with the known dormancy-associated polymorphisms in *TaMFT* and *TaMKK3*. If so, then these perfect markers can be used for selecting PHS tolerance within the breeding programs represented in this GWAS. Identifying QTL near regions of known PHS QTL validates the GWAS and suggests that breeding programs in the northwestern U.S. have historically used multiple sources of PHS tolerance.

The strong *QPHS.wsu-2D* locus co-localized both with a known PHS locus and with the *C* locus that determines club head type (Supplementary Table 3; Johnson et al., 2008). The strong *QPHS.wsu-2D* locus associated with the *C* locus may partly be an artifact because the club wheat breeding program was the dominant source of this PHS tolerance locus in the mapping panel. Since *QPHS.wsu-2D* is <1 cM from the *C* locus flanking marker wmc144, this invited the question as to whether the *C* locus itself provided PHS tolerance or whether there was another PHS-tolerance locus in tight linkage with the *C* locus. The latter seemed more likely because previous studies found that the club head type took up more water during rain events and was sometimes associated with higher preharvest sprouting in near-isogenic lines (Hong, 1979; King and Richards, 1984; R.E. Allan, personal comm.). Interestingly, there were three PHS tolerant lax wheat lines that carried the *QPHS.wsu-2D* locus (J950409-10-4, J950409-10-5, and ID581), and there were two PHS susceptible club wheat lines (J970057-5 and ARS00226) that did not carry the *QPHS.wsu-2D* locus. While these counter-examples suggest that there was a PHS QTL strongly linked to the *C* locus, they are not proof because these PHS phenotypes may have resulted from variation at other loci. Future work will need to examine this question using near-isogenic lines that differ only for the *C* locus and for the *QPHS.wsu-2D* locus.

### Breeding for PHS tolerance based on spike-wetting tests and FN

Sprouting scores for this population did not correlate strongly with FN (Table 2). While increasing number of *QPHS.wsu* loci correlated to increasing sprouting index and sprouting scores, we observed little or no correlation to increasing FN in natural rain, artificial rain, or no-rain environments (Figure 4; Supplementary Figure 6). Moreover, there was no strong association between increasing *QFN.wsu* loci and sprouting scores. In fact, sprouting scores and FN provided complementary information. It is possible that the tendency toward low FN is dependent more on the timing and strength of α-amylase induction during seed imbibition, then on the timing of visible sprout/germination *per se*. For example, there may be varieties that induce α-amylase earlier in the germination program, prior to germination *per se*. Such varieties would be prone to higher α-amylase/lower FN than expected based on the timing of visible sprout.

Breeding for FN is complicated by the fact that it is a complex trait governed by multiple factors. Although the intention was to map preharvest sprouting QTL, our FN field environments may have also experienced conditions that induced late maturity α-amylase (LMA). During LMA, α-amylase is induced in response to large temperature fluctuations during late grain filling (Farrell and Kettlewell, 2008; Mares and Mrva, 2014). While four of the five environments experienced either natural or artificial rain events, we cannot rule out the possibility that the wheat also experienced LMA. Indeed, the *QFN.wsu-7B.1* locus co-localized with a large LMA QTL (Figure 5; Mrva and Mares, 2001; McNeil et al., 2009; Emebiri et al., 2010). In the environment without rain, differences in FN likely resulted from differences in properties of grain starch and protein (Graybosch et al., 2000; Guo et al., 2003; Ross et al., 2012). Such properties likely also impact FN over 300 s when there is a rain event. The FN test has a fairly high standard deviation within technical replicates (Supplementary Figure 7). The genotypic repeatability (*R*^2^) of FN increased when we took experimental covariates (such as machine and operator) and technical replicates into account (Table 3). The FN test has other limitations for breeding such as the need for an expensive instrument, and the fact that it is more time-consuming to run FN than spike-wetting tests. Future research should examine whether α-amylase enzyme assays (Phadebas™ or Megazyme) or ELISA assays may serve as a faster, cheaper, or less variable proxy to FN (Mares and Mrva, 2008; Barrero et al., 2013). Environmental factors also caused variation in FN, resulting in only moderate correlations between environments in the current study (Table 2A). While the Zhang et al. (2014) study had higher correlations between FN in different environments (0.43 > *r* > 0.80), it had fewer samples below 300 s suggesting that less sprouting occurred in their environments. Breeders cannot rely on natural rain occurring when they want to screen for low FN due to PHS, making the use of artificial rain necessary. Other differences in the environment, such as temperature during maturation or temperature during the sprout-inducing rainfall, can impact grain dormancy, PHS susceptibility, and FN. Future artificial rain experiments may be improved by applying the artificial rain during lower evening temperatures or letting the wheat after-ripen longer in the field prior to misting.

The spike-wetting test has long been favored for selecting preharvest sprouting tolerance because the experimental design takes into account after-ripening time, spike morphology, and grain dormancy/germinability (Paterson et al., 1989). A limitation of FN testing of field-harvested grain is that differences in maturation date can be a major covariate, since early maturing varieties may have lost more dormancy through after-ripening than late maturing varieties. The spike-wetting test reduces this problem by harvesting spikes at physiological maturity and then after-ripening for the same number of days before conducting the test. When screening large numbers of breeding lines, it would be convenient to avoid scoring daily over 3 to 7 days of misting. Based on the correlations between spike-wetting test and all FN environments (with and without rain), scoring after 4 days of misting should provide breeders with both adequate variation and higher correlation to FN (Table 2B). Scoring after 4 days misting also provides good insight into initial germination capacity (scores 1-5) rather than speed of seedling growth (scores 6-10) (Figure 1). However, it should be noted that the day 6 sprouting score had the highest genotypic repeatability in this study. One drawback of selecting for PHS tolerance based on seed dormancy, is that too much dormancy may result in poor seedling emergence of winter wheat when grain is planted ~8 weeks after harvest (Rodríguez et al., 2015). Future work will need to develop a genomic selection model for breeding wheat with sufficient seed dormancy to prevent preharvest sprouting without compromising seedling emergence.

Seed dormancy and PHS tolerance are stronger if temperatures are cool during grain maturation (Nakamura et al., 2011). One could remove temperature during grain development as a variable in spike-wetting tests by growing plants in a controlled environment instead of in the field. We might have also seen better correlations if spike-wetting tests had been performed for the entire trial in the 2 years with natural rain events. However, when spike-wetting tests were performed on 162 accessions in Pullman 2013, only a −0.26 correlation was observed. A more likely explanation is that FN in these environments was impacted by multiple factors in addition to preharvest sprouting, including LMA, starch, and protein characteristics. Thus, within this study, FN and spike-wetting tests were not two ways to measure the same trait.

PHS tolerance is profoundly impacted by environmental conditions during grain maturation and during the sprout-inducing rain event (Cao et al., 2016; Kashiwakura et al., 2016; Martinez et al., 2016). Thus, it is not uncommon to find lack of agreement between environments in PHS tolerance association studies based on spike-wetting tests (Ogbonnaya et al., 2008; Jaiswal et al., 2012; Kulwal et al., 2012; Zhou et al., 2017). A total of 6 out of 34 *QPHS.wsu* were identified in at least two of the five environments. In fact, the correlations between our environments were as good as those in other spike-wetting test studies (Jaiswal et al., 2012; Kulwal et al., 2012). The fact that this study identified some QTN in multiple environments and that many of the QTN identified agreed with previous studies, suggests that this association study will lay a strong foundation for future efforts to develop genomic selection for PHS tolerance in northwestern U.S. wheat.

## Author contributions

SM, KC, and CS: Designed the experiments; SM: Conducted the experiments, analyzed the phenotypic data, performed the statistical analysis and GWAS, and constructed the visualizations; JG: Curated and filtered the genotypic data and performed the LD analysis; MH: Calculated the phenotypic variation; MH and ZZ: Provided expert guidance on performing the GWAS; KC and AC: Provided field resources; CS, KC, AC, and ZZ: Obtained funding; SM and CS: Wrote the manuscript.

### Conflict of interest statement

The authors declare that the research was conducted in the absence of any commercial or financial relationships that could be construed as a potential conflict of interest.
