# Supplementary material for: Genome-Wide Association Mapping for Tolerance to Preharvest Sprouting and Low Falling Numbers in Wheat
Source: Front Plant Sci. 2018 Feb 14;9:141. doi: 10.3389/fpls.2018.00141 (PMC5817628; doi:10.3389/fpls.2018.00141)
Supplement: Supplementary file 4 [file Table4.docx]

**Supplemental Table 4. Loci significantly associated with low FN when the maximum FN is 400 sec (FN >400 = 400) and early sprouting with the maximum sprouting score set to 5 (scores 1-5).**

| **QTL**^a^ | **Marker** | **Chr**^b^ | **cM**^b^ | **-log10(*p*)** | **maf** | **Effect** ^c^ | ***r^2^*** | **Environment** | **Favorable Allele** ^d^ |
| --- | --- | --- | --- | --- | --- | --- | --- | --- | --- |
| ***QFN.wsu-6A*** | IWB12127 | 6A | 37 | 6.24 | 0.05 | 17.1 | 0.013 | C16 FN | **A**/G |
| ***QFN.wsu-7A.3*** | IWB73683 | 7A | 61.8 | 6.61 | 0.11 | 13.3 | 0.015 | C16 FN | **A**/G |
| ***QPHSg.wsu-1A.2*** | IWB44485 | 1A | 76 | 7.05 | 0.49 | -0.06 | 0.20 | P15 d3 | **T**/G |
| ***QPHSg.wsu-1D.1*** | IWB5944 | 1D | 67 | 7.32 | 0.07 | -0.04 | 0.00 | P14 d3 | T/**C** |
| *QPHSg.wsu-2B.1* | IWB75872 | 2B | 88 | 8.87 | 0.07 | -0.04 | 0.00 | P14 d3 | A/**G** |
| *QPHSg.wsu-2D* | IWB81540 | 2D | 50 | 9.84 | 0.46 | -0.31 | 0.00 | P16 d3 | **A**/G |
|  | IWB81540 | 2D | 50 | 9.44 | 0.46 | -0.23 | 0.01 | P16 d4 | **A**/G |
|  | IWB7652 | 2D | 52 | 27.42 | 0.37 | -0.72 | 0.02 | C14 d4 | T/**C** |
| *QPHSg.wsu-3B.2* | IWB48693 | 3B | 62 | 6.71 | 0.47 | -0.20 | 0.00 | C14 d4 | **A**/G |
|  | IWB54142 | 3B | 62 | 8.82 | 0.49 | -0.17 | 0.00 | C15 d3 | A/**G** |
| *QPHSg.wsu-4A.1* | IWB80864 | 4A | 58 | 10.84 | 0.05 | -0.08 | 0.00 | P14 d3 | A/**G** |
| ***QPHSg.wsu-4A.2*** | IWB61756 | 4A | 109 | 7.04 | 0.49 | -0.06 | 0.00 | P15 d3 | **A**/G |
| *QPHSg.wsu-5B.1* | IWB33287 | 5B | 19 | 10.40 | 0.38 | -0.19 | 0.04 | P15 d4 | A/**G** |
| ***QPHSg.wsu-5B.2*** | IWB22696 | 5B | 40 | 7.01 | 0.25 | -0.05 | 0.02 | P15 d3 | A/**G** |
| ***QPHSg.wsu-5B.3*** | IWB33771 | 5B | 117 | 7.10 | 0.43 | -0.25 | 0.01 | P14 d4 | **A**/G |
| ***QPHSg.wsu-6A.3*** | IWB40151 | 6A | 85 | 11.40 | 0.48 | -0.07 | 0.00 | P15 d3 | A/**G** |
| ***QPHSg.wsu-7A.2*** | IWB34499 | 7A | 97 | 7.61 | 0.39 | -0.29 | 0.00 | C15 d4 | A/**C** |
|  | IWB59295 | 7A | 97 | 8.82 | 0.39 | -0.20 | 0.01 | P15 d4 | A/**G** |
| ***QPHSg.wsu-7A.5*** | IWB79354 | 7A | 203 | 8.00 | 0.24 | -0.07 | 0.20 | P15 d3 | A/**G** |
| ***QPHSg.wsu-7A.6*** | IWB26780 | 7A | 216 | 7.25 | 0.34 | -0.20 | 0.19 | C14 d4 | **T**/C |
| ***QPHSg.wsu-1B*** | IWB63380 | 1B | 90 | 7.29 | 0.11 | -0.22 | 0.03 | P15 d5 | A/**G** |
| *QPHSg.wsu-1D.2* | IWB71680 | 1D | 163 | 9.35 | 0.06 | -0.24 | 0.00 | P16 d5 | **A**/G |
| *QPHSg.wsu-2D* | IWB46396 | 2D | 54 | 29.29 | 0.39 | -0.54 | 0.10 | C14 d5 | **A**/G |
| ***QPHSg.wsu-3A.4*** | IWB8288 | 3A | 151 | 7.57 | 0.12 | -0.16 | 0.00 | P16 d5 | A/**C** |
| *QPHSg.wsu-3B.3* | IWB8629 | 3B | 67 | 7.11 | 0.08 | -0.15 | 0.00 | P16 d5 | A/**G** |
| ***QPHSg.wsu-4D*** | IWB10053 | 4D | 79 | 7.20 | 0.09 | -0.29 | 0.05 | P15 d5 | T/**C** |
| ***QPHSg.wsu-5A.1*** | IWB10250 | 5A | 70 | 8.07 | 0.32 | -0.27 | 0.01 | P15 d5 | **T**/C |
| *QPHSg.wsu-6A.2* | IWB51108 | 6A | 77 | 6.97 | 0.34 | -0.15 | 0.09 | P14 d5 | **A**/G |
| *QPHSg.wsu-7B.2* | IWB7099 | 7B | 133 | 6.57 | 0.17 | -0.21 | 0.00 | C14 d5 | A/**G** |
| ***QPHSg.wsu-unk.2*** | IWB12564 | unk | - | 8.64 | 0.06 | -0.39 | 0.00 | P15 d5 | T/**C** |
| ***QPHSg.wsu-unk.3*** | IWB74624 | unk | - | 6.74 | 0.18 | -0.10 | 0.03 | P16 d5 | A/**G** |
| ***QPHSg.wsu-1A.1*** | IWB9223 | 1A | 16 | 7.58 | 0.18 | -0.09 | 0.02 | P15 d7 | T/**C** |
| *QPHSg.wsu-1D.2* | IWB71680 | 1D | 163 | 8.29 | 0.06 | -0.14 | 0.00 | P14 d7 | **A**/G |
|  | IWB71680 | 1D | 163 | 13.58 | 0.06 | -0.19 | 0.02 | P16 d6 | **A**/G |
| *QPHSg.wsu-2A.1* | IWB17580 | 2A | 53 | 11.58 | 0.07 | -0.29 | 0.01 | C15 d6 | T/**C** |
|  | IWB17580 | 2A | 53 | 14.21 | 0.07 | -0.28 | 0.01 | C15 d7 | T/**C** |
| ***QPHSg.wsu-2A.2*** | IWB64379 | 2A | 128 | 6.96 | 0.08 | -0.17 | 0.00 | P14 d6 | **A**/G |
| *QPHSg.wsu-2B.1* | IWB27957 | 2B | 92 | 11.90 | 0.06 | -0.13 | 0.02 | P16 d6 | A/**G** |
| ***QPHSg.wsu-2B.3*** | IWB64082 | 2B | 141 | 9.54 | 0.10 | -0.14 | 0.07 | C14 d7 | **A**/G |
| *QPHSg.wsu-2D* | IWB7652 | 2D | 52 | 18.90 | 0.37 | -0.39 | 0.11 | C14 d6 | T/**C** |
|  | IWB7652 | 2D | 52 | 15.04 | 0.37 | -0.21 | 0.07 | C14 d7 | T/**C** |
| *QPHSg.wsu-3A.1* | IWB50475 | 3A | 15 | 6.65 | 0.33 | -0.11 | 0.01 | C14 d6 | **A**/G |
| ***QPHSg.wsu-3A.2*** | IWB32758 | 3A | 113 | 7.53 | 0.07 | -0.15 | 0.02 | P16 d6 | **T**/G |
| *QPHSg.wsu-3B.1* | IWB7629 | 3B | 5 | 8.85 | 0.07 | -0.14 | 0.00 | P14 d7 | T/**G** |
| *QPHSg.wsu-3B.3* | IWB45058 | 3B | 67 | 7.62 | 0.10 | -0.11 | 0.00 | P16 d6 | A/**G** |
| ***QPHSg.wsu-3B.4*** | IWB70167 | 3B | 137 | 7.86 | 0.05 | -0.15 | 0.04 | P16 d6 | A/**G** |
| *QPHSg.wsu-5A.2* | IWB6049 | 5A | 84 | 7.20 | 0.19 | -0.07 | 0.00 | P16 d6 | **A**/G |
| ***QPHSg.wsu-5B.4*** | IWB71749 | 5B | 144 | 9.99 | 0.11 | -0.28 | 0.01 | C15 d6 | **A**/G |
|  | IWB71749 | 5B | 144 | 17.54 | 0.11 | -0.36 | 0.00 | C15 d7 | **A**/G |
| *QPHSg.wsu-6D* | IWB49280 | 6D | 153 | 7.10 | 0.09 | -0.22 | 0.00 | P15 d6 | **A**/G |
| ***QPHSg.wsu-7A.1*** | IWB47598 | 7A | 45 | 7.58 | 0.45 | -0.07 | 0.04 | P16 d6 | A/**G** |
| ***QPHSg.wsu-7A.3*** | IWB54820 | 7A | 178 | 6.65 | 0.46 | -0.07 | 0.00 | P14 d7 | **A**/G |
| ***QPHSg.wsu-7B.1*** | IWB78079 | 7B | 77 | 8.29 | 0.07 | -0.04 | 0.00 | P14 d7 | **A**/G |
|  | IWB81211 | 7B | 77 | 7.87 | 0.06 | -0.35 | 0.02 | P14 d7 | A/**G** |
|  | IWB59498 | 7B | 77 | 7.87 | 0.06 | -0.35 | 0.00 | P14 d7 | **A**/G |
|  | IWB25969 | 7B | 78 | 8.11 | 0.06 | -0.37 | 0.00 | P14 d7 | **A**/G |
|  | IWB26968 | 7B | 78 | 8.11 | 0.06 | -0.37 | 0.00 | P14 d7 | **A**/G |
|  | IWB26969 | 7B | 78 | 8.11 | 0.06 | -0.37 | 0.01 | P14 d7 | **A**/G |
| ***QPHSg.wsu-7D*** | IWB7177 | 7D | 149 | 7.75 | 0.45 | -0.09 | 0.01 | C14 d7 | **A**/G |
| ***QPHSg.wsu-unk.1*** | IWB12177 | unk | - | 10.02 | 0.32 | -0.21 | 0.01 | C15 d6 | A/**G** |
| ***QPHSg.wsu-unk.4*** | IWB50340 | unk | - | 8.12 | 0.09 | -0.18 | 0.00 | P16 d6 | **A**/G |
| ***QPHSg.wsu-2B.2*** | IWB63900 | 2B | 104 | 7.77 | 0.22 | -0.02 | 0.00 | C14 SI | A/**G** |
| *QPHSg.wsu-2D* | IWB81540 | 2D | 50 | 7.64 | 0.46 | -0.02 | 0.03 | P16 SI | **A**/G |
|  | IWB7652 | 2D | 52 | 37.13 | 0.37 | -0.06 | 0.00 | C14 SI | T/**C** |
| ***QPHSg.wsu-3A.3*** | IWB46039 | 3A | 138 | 8.17 | 0.08 | -0.03 | 0.00 | C14 SI | A/**G** |
| *QPHSg.wsu-5A.1* | IWB10250 | 5A | 70 | 9.33 | 0.32 | -0.02 | 0.16 | P15 SI | **T**/C |
| ***QPHSg.wsu-5A.3*** | IWB66227 | 5A | 96 | 6.55 | 0.43 | -0.02 | 0.03 | C15 SI | T/**C** |
| ***QPHSg.wsu-6A.1*** | IWB80412 | 6A | 5 | 8.38 | 0.22 | -0.02 | 0.00 | C14 SI | **A**/G |
| ***QPHSg.wsu-7A.2*** | IWB59295 | 7A | 97 | 10.04 | 0.39 | -0.03 | 0.02 | C15 SI | A/**G** |
| ***QPHSg.wsu-7A.4*** | IWB29877 | 7A | 181 | 7.23 | 0.10 | -0.02 | 0.01 | C14 SI | **T**/C |

^a^ QTL in bold are not found in the original GWAS analysis (Table 4) assuming QTN within 10cM are the same QTL.

^b^ Chromosome and position according to Wang et al. (2014).

^c^ The allelic effect is shown in FN seconds or sprouting score BLUPs.

^d^ The significant allele is favorable (in bold) if it decreases sprouting scores in the spike wetting tests or increases Falling Numbers.
