# Supplementary material for: Genome-Wide Association Mapping for Tolerance to Preharvest Sprouting and Low Falling Numbers in Wheat
Source: Front Plant Sci. 2018 Feb 14;9:141. doi: 10.3389/fpls.2018.00141 (PMC5817628; doi:10.3389/fpls.2018.00141)
Supplement: Supplementary file 5 [file Table5.DOCX]

**Supplemental Table 5. Loci significantly associated with heading date (HD) from Pullman 2014.**

| **QTL**^a^ | **Marker** | **Chr** ^b^ | **cM** ^b^ | **-log10(*p*)** | **maf** | **Effect** ^c^ | ***r^2^*** | **Environment** | **Later Allele** ^d^ |
| --- | --- | --- | --- | --- | --- | --- | --- | --- | --- |
| *QHD.wsu-1B.1* | IWB64963 | 1B | 60 | 12.83 | 0.05 | 0.99 | 0.09 | CF16 FN | A/**G** |
| *QHD.wsu-1B.2* | IWB68096 | 1B | 160 | 9.39 | 0.33 | 0.46 | 0.00 | PUL15 FN | **A**/G |
| *QHD.wsu-2B* | IWB37419 | 2B | 91 | 7.26 | 0.34 | 0.44 | 0.00 | PUL13 | **T**/C |
| *QHD.wsu-4A* | IWB1396 | 4A | 41 | 11.29 | 0.15 | 0.56 | 0.01 | PUL15 FN | **T**/G |

^a^ QTL in bold explained 10% (R^2^ >0.1) or more of the phenotypic variation.

^b^ Chromosome and position according to Wang et al. (2014).

^c^ The allelic effect is in days.

^d^ The significant allele which causes later heading date is highlighted in bold.
