# Supplementary material for: Genome-Wide Association Mapping for Tolerance to Preharvest Sprouting and Low Falling Numbers in Wheat
Source: Front Plant Sci. 2018 Feb 14;9:141. doi: 10.3389/fpls.2018.00141 (PMC5817628; doi:10.3389/fpls.2018.00141)
Supplement: Supplementary file 6 [file Table6.DOCX]

**Supplemental Table 6. Loci significantly associated with FN, sprouting (PHS) scores across days (d) 3-7, and sprouting index (SI) with no principal components (nPC) incorporated into the association mapping model.**

| **QTL**^a^ | **Marker** | **Chr**^b^ | **cM**^b^ | **-log10(*p*)** | **maf** | **Effect** ^c^ | ***r^2^*** | **Environment** | **Favorable Allele** ^d^ |
| --- | --- | --- | --- | --- | --- | --- | --- | --- | --- |
| ***QFNnPC.wsu-1B*** | IWB24969 | 1B | 75 | 8.62 | 0.12 | 10.07 | 0.00 | P15 | **T**/C |
| ***QFNnPC.wsu-4A.1*** | IWB59982 | 4A | 54 | 8.57 | 0.12 | 16.11 | 0.00 | C16 | A/**G** |
| ***QFNnPC.wsu-4A.2*** | IWB59774 | 4A | 164 | 7.01 | 0.07 | 21.46 | 0.00 | C15 | **A**/G |
| *QFNnPC.wsu-7A* | IWB14901 | 7A | 124 | 6.69 | 0.18 | 15.85 | 0.02 | C15 | **T**/C |
|  | IWB75568 | 7A | 126 | 13.88 | 0.41 | 8.19 | 0.03 | P15 | A/**C** |
| ***QPHSnPC.wsu-1A.1*** | IWB34707 | 1A | 16 | 6.81 | 0.41 | -0.36 | 0.15 | P14 d4 | A/**C** |
| ***QPHSnPC.wsu-1A.2*** | IWB44485 | 1A | 76 | 7.06 | 0.49 | -0.06 | 0.00 | P15 d3 | **T**/G |
| *QPHSnPC.wsu-1A.3* | IWB77080 | 1A | 87 | 7.25 | 0.35 | -0.02 | 0.00 | C15 SI | A/**G** |
| *QPHSnPC.wsu-1B.1* | IWB22868 | 1B | 31 | 8.04 | 0.18 | -0.34 | 0.14 | P14 d5 | **T**/C |
| ***QPHSnPC.wsu-1B.2*** | IWB47637 | 1B | 67 | 6.63 | 0.21 | -0.28 | 0.06 | C15 d4 | A/**G** |
|  | IWB12334 | 1B | 70 | 6.85 | 0.14 | -0.33 | 0.06 | P14 d5 | **A**/G |
| ***QPHSnPC.wsu-1B.3*** | IWB49534 | 1B | 81 | 7.13 | 0.46 | -0.14 | 0.02 | C15 d3 | A/**G** |
| *QPHSnPC.wsu-1B.4* | IWB63380 | 1B | 90 | 10.18 | 0.11 | -0.34 | 0.01 | P15 d5 | A/**G** |
|  | IWB77753 | 1B | 90 | 6.84 | 0.10 | -1.05 | 0.00 | P15 d5 | **A**/G |
| ***QPHSnPC.wsu-1D.1*** | IWB5944 | 1D | 67 | 7.32 | 0.07 | -0.04 | 0.00 | P14 d3 | T/**C** |
| *QPHSnPC.wsu-1D.2* | IWB71680 | 1D | 163 | 12.65 | 0.06 | -0.64 | 0.01 | P16 d6 | **A**/G |
|  | IWB71680 | 1D | 163 | 15.54 | 0.06 | -0.61 | 0.00 | P16 d7 | **A**/G |
| ***QPHSnPC.wsu-2A.1*** | IWB26001 | 2A | 150 | 7.19 | 0.16 | -0.02 | 0.31 | C14 SI | **T**/C |
| *QPHSnPC.wsu-2B.1* | IWB75872 | 2B | 88 | 8.87 | 0.07 | -0.04 | 0.01 | P14 d3 | A/**G** |
|  | IWB33642 | 2B | 90 | 7.01 | 0.10 | -0.37 | 0.08 | C14 d5 | **A**/G |
| ***QPHSnPC.wsu-2B.2*** | IWB63970 | 2B | 114 | 6.62 | 0.36 | -0.27 | 0.08 | C14 d6 | A/**G** |
|  | IWB63970 | 2B | 114 | 7.29 | 0.36 | -0.02 | 0.00 | C14 SI | A/**G** |
| *QPHSnPC.wsu-2D* | IWB81540 | 2D | 50 | 9.84 | 0.46 | -0.31 | 0.01 | P16 d3 | **A**/G |
|  | IWB81540 | 2D | 50 | 7.05 | 0.46 | -0.26 | 0.07 | P16 d4 | **A**/G |
|  | IWB7652 | 2D | 52 | 23.74 | 0.37 | -0.69 | 0.00 | C14 d4 | T/**C** |
|  | IWB7652 | 2D | 52 | 25.31 | 0.37 | -1.08 | 0.10 | C14 d6 | T/**C** |
|  | IWB46396 | 2D | 54 | 30.03 | 0.39 | -0.82 | 0.24 | C14 d5 | **A**/G |
|  | IWB46396 | 2D | 54 | 11.44 | 0.39 | -0.61 | 0.08 | C14 d7 | **A**/G |
|  | IWB46396 | 2D | 54 | 23.14 | 0.39 | -0.04 | 0.17 | C14 SI | **A**/G |
| *QPHSnPC.wsu-3A.1* | IWB60440 | 3A | 15 | 7.52 | 0.47 | -0.30 | 0.01 | P14 d7 | A/**G** |
| ***QPHSnPC.wsu-3A.2*** | IWB78290 | 3A | 89 | 6.80 | 0.41 | -0.29 | 0.08 | P15 d5 | A/**G** |
| ***QPHSnPC.wsu-3A.3*** | IWB78959 | 3A | 105 | 9.81 | 0.19 | -0.44 | 0.01 | C14 d6 | A/**G** |
| ***QPHSnPC.wsu-3A.4*** | IWB8288 | 3A | 151 | 6.56 | 0.12 | -0.36 | 0.00 | P16 d6 | A/**C** |
|  | IWB8288 | 3A | 151 | 7.64 | 0.12 | -0.18 | 0.03 | P16 d7 | A/**C** |
| *QPHSnPC.wsu-3B.1* | IWB54142 | 3B | 62 | 8.40 | 0.49 | -0.16 | 0.02 | C15 d3 | A/**G** |
|  | IWB36421 | 3B | 62 | 7.00 | 0.46 | -0.26 | 0.00 | P14 d6 | **A**/G |
|  | IWB36421 | 3B | 62 | 8.10 | 0.46 | -0.01 | 0.00 | P14 SI | **A**/G |
|  | IWB79762 | 3B | 62 | 8.92 | 0.47 | -0.29 | 0.00 | P16 d6 | A/**G** |
| ***QPHSnPC.wsu-3B.2*** | IWB8629 | 3B | 67 | 7.46 | 0.08 | -0.52 | 0.01 | P16 d6 | A/**G** |
| ***QPHSnPC.wsu-3B.3*** | IWB10839 | 3B | 139 | 8.95 | 0.40 | -0.33 | 0.00 | P16 d6 | **T**/G |
| ***QPHSnPC.wsu-3D*** | IWB27554 | 3D | 155 | 6.77 | 0.05 | -0.51 | 0.07 | P15 d5 | **A**/G |
| *QPHSnPC.wsu-4A.1* | IWB80864 | 4A | 58 | 10.85 | 0.05 | -0.08 | 0.01 | P14 d3 | A/**G** |
| ***QPHSnPC.wsu-4A.2*** | IWB61756 | 4A | 109 | 7.04 | 0.49 | -0.06 | 0.00 | P15 d3 | **A**/G |
| *QPHSnPC.wsu-4B.1* | IWB8210 | 4B | 61 | 9.86 | 0.09 | -0.57 | 0.01 | P14 d7 | A/**G** |
| *QPHSnPC.wsu-4B.2* | IWB76214 | 4B | 73 | 7.60 | 0.06 | -0.48 | 0.00 | P15 d7 | A/**G** |
| ***QPHSnPC.wsu-4D*** | IWB10053 | 4D | 79 | 9.21 | 0.09 | -0.53 | 0.02 | P15 d5 | T/**C** |
| *QPHSnPC.wsu-5A.1* | IWB10998 | 5A | 53 | 8.74 | 0.40 | -0.02 | 0.26 | C14 SI | T/**C** |
| *QPHSnPC.wsu-5A.2* | IWB10250 | 5A | 70 | 8.61 | 0.32 | -0.40 | 0.01 | P15 d5 | **T**/C |
|  | IWB76988 | 5A | 73 | 6.95 | 0.33 | -0.26 | 0.00 | P14 d5 | A/**G** |
|  | IWB76988 | 5A | 73 | 6.69 | 0.33 | -0.01 | 0.01 | P14 SI | A/**G** |
| *QPHSnPC.wsu-5A.3* | IWB6049 | 5A | 84 | 8.42 | 0.19 | -0.29 | 0.01 | P16 d6 | **A**/G |
|  | IWB40144 | 5A | 86 | 7.27 | 0.41 | -0.37 | 0.00 | P15 d6 | **A**/G |
| ***QPHSnPC.wsu-5A.4*** | IWB66227 | 5A | 96 | 7.46 | 0.43 | -0.29 | 0.01 | C15 d4 | T/**C** |
| ***QPHSnPC.wsu-5B.1*** | IWB22696 | 5B | 40 | 7.02 | 0.25 | -0.05 | 0.01 | P15 d3 | A/**G** |
| ***QPHSnPC.wsu-5B.2*** | IWB56759 | 5B | 60 | 7.27 | 0.05 | -0.31 | 0.00 | C15 d3 | **A**/G |
| ***QPHSnPC.wsu-5B.3*** | IWB71749 | 5B | 144 | 10.33 | 0.11 | -0.83 | 0.00 | C15 d7 | **A**/G |
| ***QPHSnPC.wsu-5D*** | IWB81433 | 5D | 60 | 8.70 | 0.47 | -0.34 | 0.00 | P14 d7 | **A**/G |
| ***QPHSnPC.wsu-6A.1*** | IWB40151 | 6A | 85 | 11.40 | 0.48 | -0.07 | 0.00 | P15 d3 | A/**G** |
| ***QPHSnPC.wsu-6A.2*** | IWB3818 | 6A | 130 | 6.96 | 0.21 | -0.24 | 0.02 | C14 d4 | **T**/C |
| *QPHSnPC.wsu-6D* | IWB49280 | 6D | 153 | 7.27 | 0.09 | -0.36 | 0.03 | P15 d7 | **A**/G |
| ***QPHSnPC.wsu-7A.1*** | IWB36251 | 7A | 33 | 6.70 | 0.28 | -0.14 | 0.03 | P15 d5 | **A**/C |
| ***QPHSnPC.wsu-7A.2*** | IWB34499 | 7A | 97 | 6.77 | 0.39 | -0.30 | 0.04 | C15 d4 | A/**C** |
|  | IWB59295 | 7A | 97 | 9.58 | 0.39 | -0.27 | 0.00 | P15 d4 | A/**G** |
|  | IWB22776 | 7A | 97 | 7.43 | 0.40 | -0.36 | 0.01 | P15 d5 | **T**/C |
| ***QPHSnPC.wsu-7A.3*** | IWB79354 | 7A | 203 | 8.00 | 0.24 | -0.08 | 0.00 | P15 d3 | A/**G** |
| ***QPHSnPC.wsu-7A.4*** | IWB25497 | 7A | 230 | 8.58 | 0.12 | -0.46 | 0.00 | P15 d5 | **A**/G |
| ***QPHSnPC.wsu-7B.1*** | IWB25434 | 7B | 24 | 10.26 | 0.08 | -0.52 | 0.04 | P16 d7 | T/**G** |
| *QPHSnPC.wsu-7B.2* | IWB7099 | 7B | 133 | 9.92 | 0.17 | -0.57 | 0.03 | C14 d7 | A/**G** |
| ***QPHSnPC.wsu-unk*** | IWB71903 | unk | - | 7.91 | 0.28 | -0.37 | 0.02 | P15 d5 | A/**G** |
| ***QPHSnPC.wsu-unk*** | IWB71903 | unk | - | 6.79 | 0.28 | -0.02 | 0.01 | P15 SI | A/**G** |
| ***QPHSnPC.wsu-unk*** | IWB7559 | unk | - | 7.31 | 0.40 | -0.34 | 0.01 | P16 d5 | T/**C** |
| ***QPHSnPC.wsu-unk*** | IWB65338 | unk | - | 9.17 | 0.05 | -0.39 | 0.04 | P16 d7 | A/**G** |
| ***QPHSnPC.wsu-unk*** | IWB35677 | unk | - | 7.22 | 0.13 | -0.64 | 0.03 | P16 d7 | A/**C** |

^a^ QTL in bold are not found in the original GWAS analysis (Table 4) assuming QTN within 10cM are the same QTL.

^b^ Chromosome and position according to Wang et al. (2014).

^c^ The allelic effect is shown in FN seconds or sprouting score BLUPs.

^d^ The significant allele is favorable (in bold) if it decreases sprouting scores in the spike wetting tests or increases Falling Numbers.
