# Supplementary material for: Genome-Wide Association Mapping for Tolerance to Preharvest Sprouting and Low Falling Numbers in Wheat
Source: Front Plant Sci. 2018 Feb 14;9:141. doi: 10.3389/fpls.2018.00141 (PMC5817628; doi:10.3389/fpls.2018.00141)
Supplement: Supplementary file 7 [file Image1.pdf]

Genome-wide Association Mapping for Tolerance to Preharvest Sprouting and Low Falling Numbers in Wheat. *Frontiers in Plant Science*

Shantel A. Martinez[1,3], Jayfred Godoy[3], Meng Huang[3], Zhiwu Zhang[1,3], Arron H. Carter[1,3], Kimberly A. Garland Campbell[1,2,3,*], and Camille M. Steber[1,2,3,*]

[1] Molecular Plant Sciences, Washington State University, Pullman, WA, USA
[2] USDA-ARS Wheat Health, Genetics and Quality Research Unit, Washington State University, Pullman, WA, USA
[3] Department of Crop and Soil Sciences, Washington State University, Pullman, WA, USA
[*] Correspondence: Camille M. Steber and Kimberly Garland Campbell
Email: camille.steber@ars.usda.gov and Kim.Garland-Campbell@ars.usda.gov

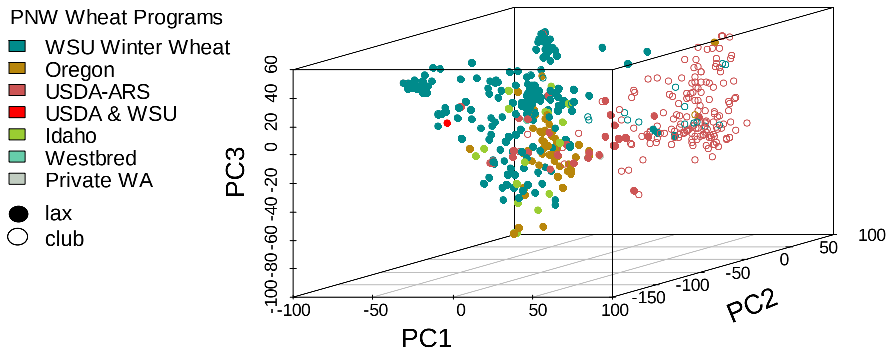

Supplemental Figure 1 | Principle components. The mapping panel was derived from at least six soft white winter wheat breeding programs and was comprised of three principle genetic components. Note that most of the club wheat (open circle) came from the USDA-ARS program.

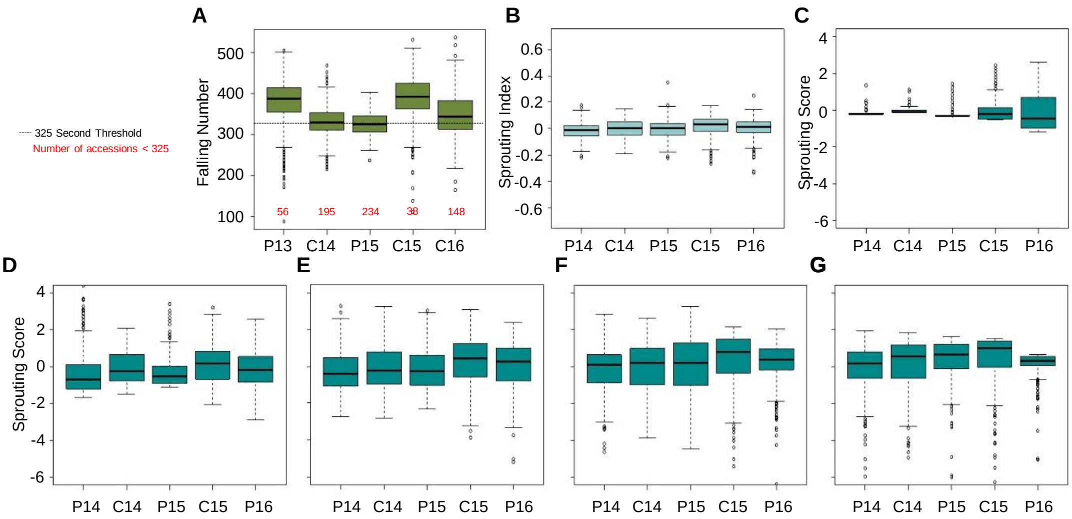

Supplemental Figure 2 | FN and sprouting score distributions. GWAS was performed using the FN least square means and the spike-wetting test BLUPs. (**A**) FN distributions across environments including the natural rain events in Pullman 2013 (P13) and Central Ferry 2016 (C16), an artificial rain event in Central Ferry 2014 (C14) and 2015 (C15), and an environment without rain in Pullman 2015 (P15). Spike-wetting tests were performed for Pullman 2014 (P14) as well as C14, P15, C15, and C16. (**B**) Sprouting index was calculated over all 7 days of misting. Sprouting scores after (**C**) 3, (**D**) 4, (**E**) 5, (**F**) 6, and (**G**) 7 days of misting.

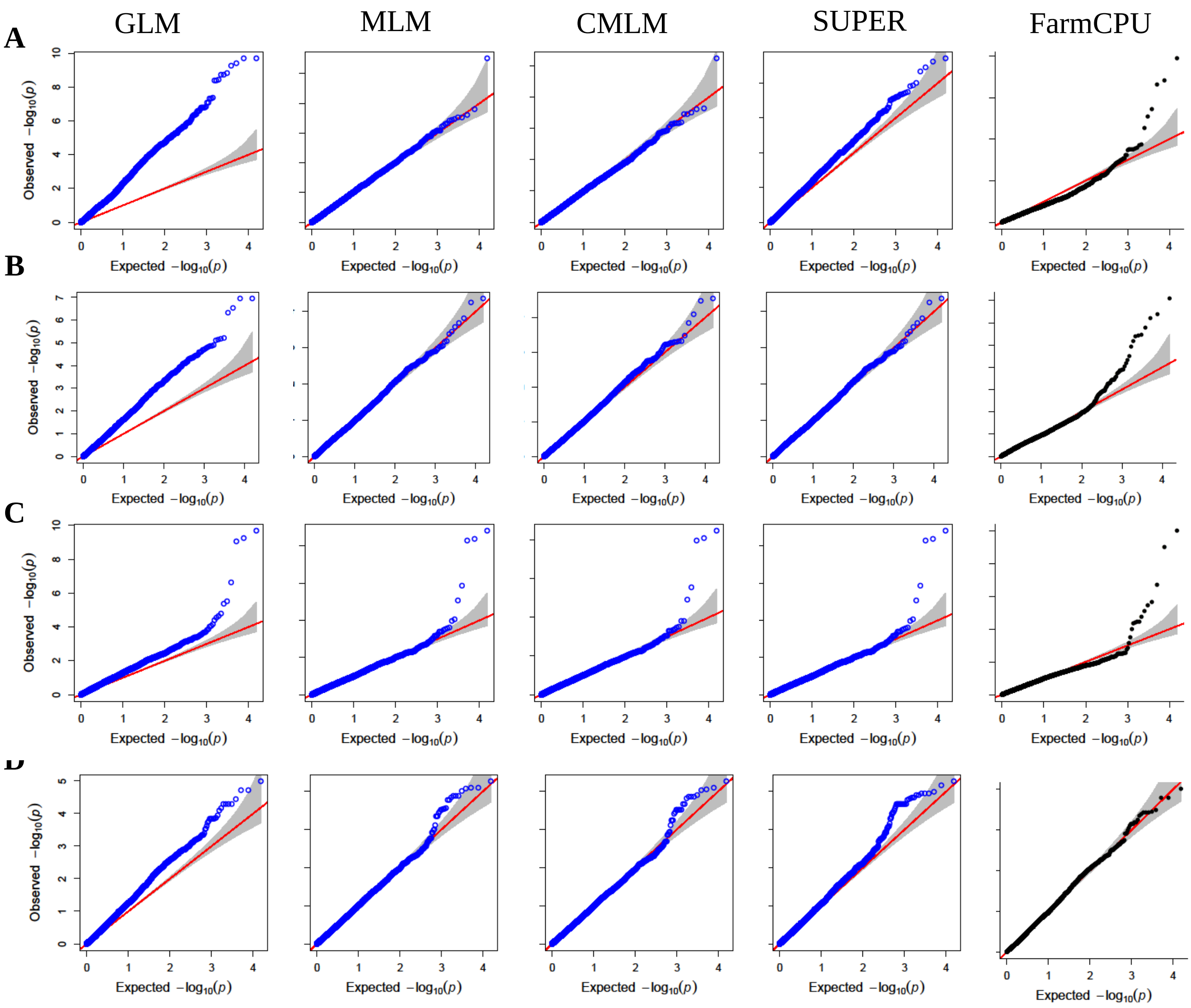

Supplemental Figure 3 | Comparison of association mapping models. Quantile-Quantile plots from GLM, MLM, CMLM, SUPER, and FarmCPU models are compared for environments: (**A**) Central Ferry 2016 Falling Numbers, (**B**) Pullman 2015 sprouting scores, (**C**) Central Ferry 2014 sprouting scores, and (**D**) Central Ferry 2015 sprouting scores. The grey region shows the 95% confidence interval for the QQ-plot under the null hypothesis of no association between the SNP marker (black or blue dot) and the trait. The FarmCPU model showed an excellent fit of observed and expected $-\log_{10}(p)$ values except for the extreme markers (upper right) that were used to make marker-trait associations.

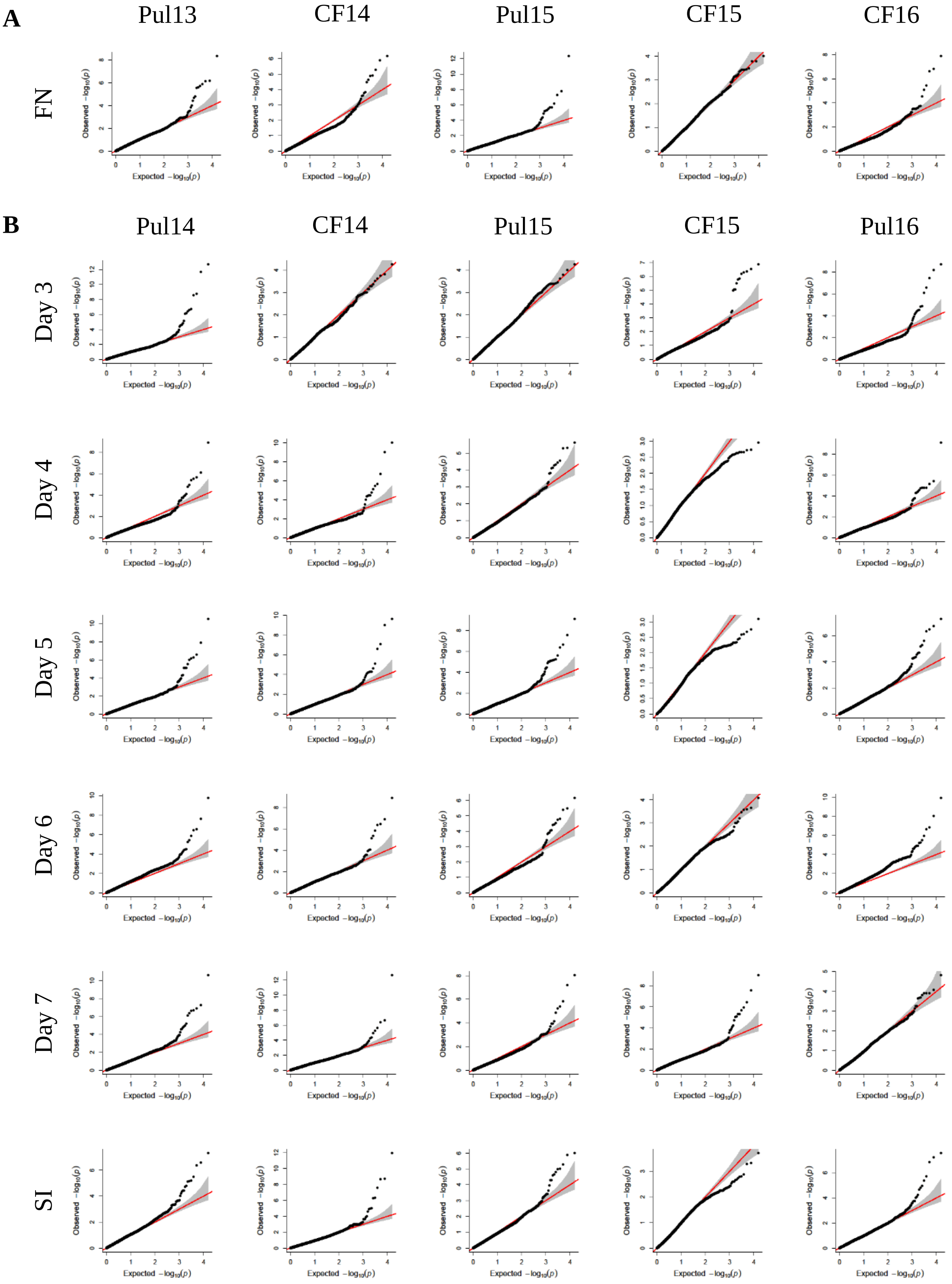

Supplemental Figure 4 | Quantile-Quantile plots of the observed versus the expected -$\log_{10}(p)$ using the FarmCPU model. QQ-plots of (**A**) Falling Number environments and (**B**) sprouting scores across different days of misting and SI environments. The grey region shows the 95% confidence interval for the QQ-plot under the null hypothesis of no association between the SNP marker (black dot) and the trait.

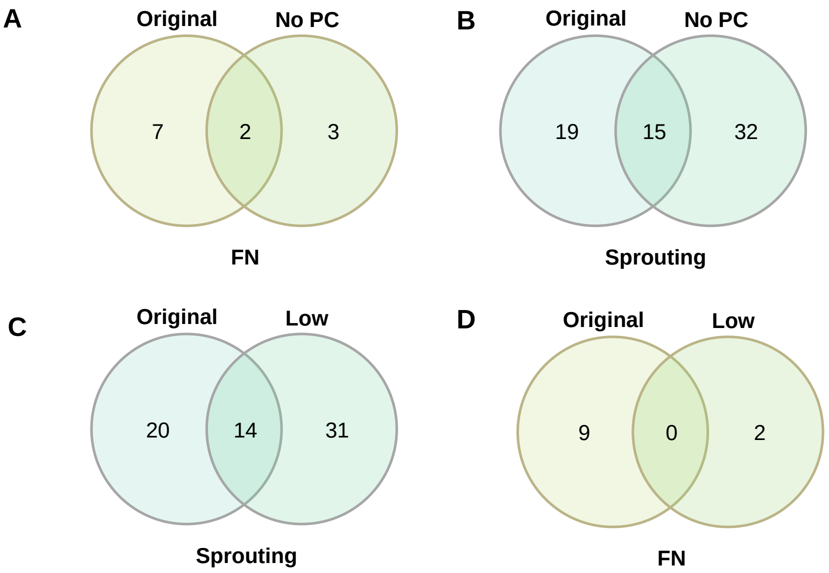

Supplemental Figure 5 | Venn Diagrams comparing significant QTN between (**A**) the original GWAS *QFN.wsu* loci versus the GWAS without using principal components for *QFNnPC.wsu* loci, (**B**) the original GWAS *QPHS.wsu* loci versus the GWAS without using principal components for *QPHSnPC.wsu* loci, (**C**) the original sprouting GWAS *QPHS.wsu* loci versus the low sprouting score GWAS *QPHSg.wsu* loci (score range only 1-5), and (**D**) the *QFN.wsu* loci from the original GWAS versus the GWAS in which the maximum FN was set to 400 sec. QTN within 10 cM region of each other based on the Wang et al. (2014) consensus map were considered to be the same QTL.

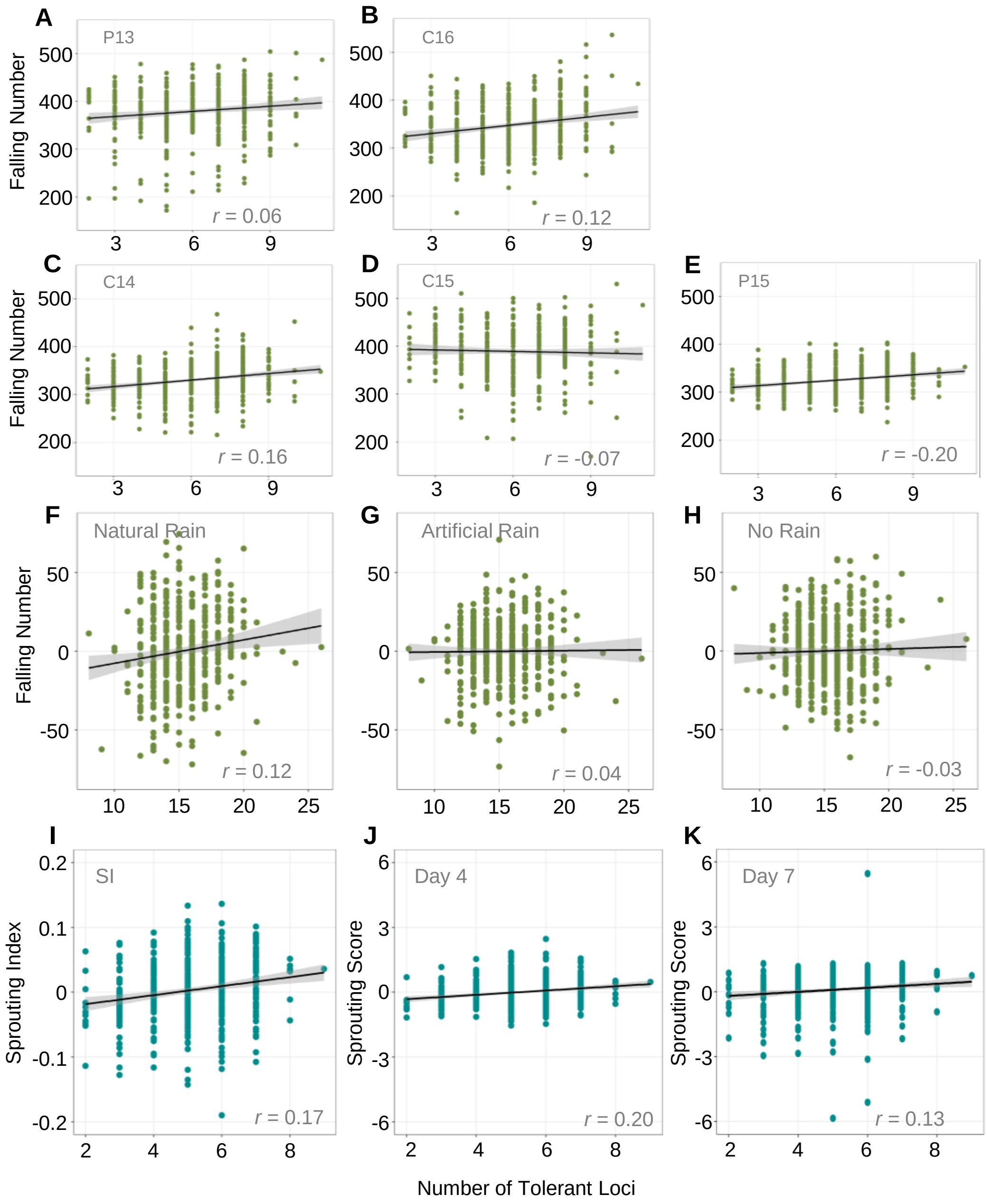

Supplemental Figure 6 | The pyramiding effect of significant *QPHS.wsu* and *QFN.wsu* loci against FN and sprouting scores. Scatter plots of the number of tolerant *QFN.wsu* loci and the FN least squared means across (**A**) Pullman 2013, (**B**) Central Ferry 2016, (**C**) Central Ferry 2014, (**D**) Central Ferry 2015, and (**E**) Pullman 2015. Tolerant *QPHS.wsu* loci were also compared to FN BLUPs across (**F**) the natural rain environments, (**G**) the artificial rain environments, and (**H**) in the absence of rain. Scatter plots of the number of tolerant *QFN.wsu* loci against (**I**) sprouting index BLUPs, (**J**) sprouting score BLUPs after 3 and (**K**) 7 days of misting. *r* is the Pearson correlation coefficient between the trait and number of tolerant loci.

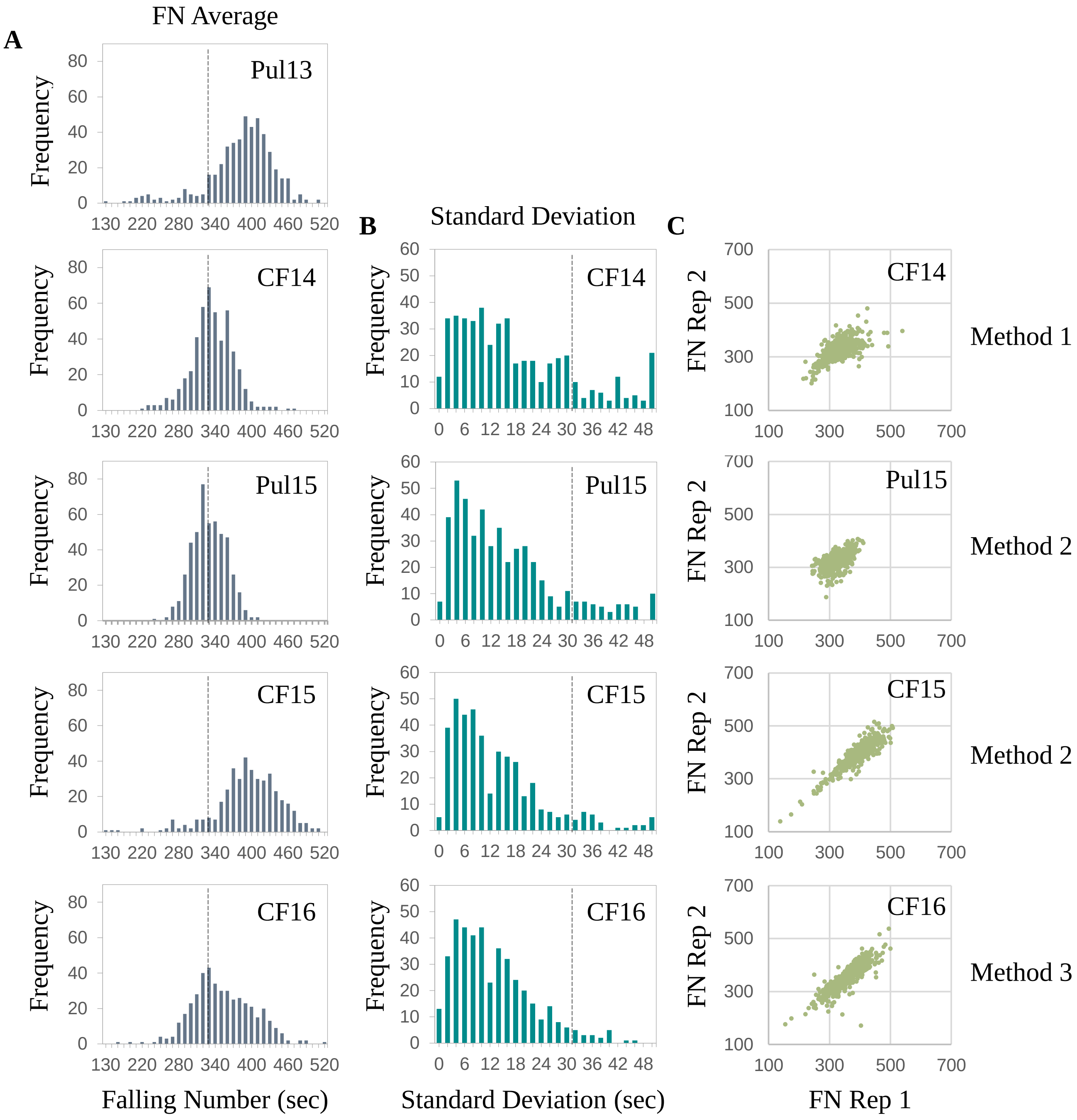

Supplemental Figure 7 | FN technical replicate reproducibility. (**A**) Histograms of FN across environments. The dotted line indicates 330 seconds, approximately the FN when we see α–amylase activity. (**B**) Histograms of the standard deviation per technical replicate across environments. The dotted line indicates 30 seconds, the expected standard deviation from the Hagberg-Perten Falling Numbers test. (**C**) Technical replicate 1 versus replicate 2 across the environments. Replicates 1 and 2 were tested on different days (method 1), within 5 minutes of each other (method 2), or side-by-side (method 3). Pullman 2013 had only 1 technical replicate.
